# Supplementary material for: Seasonal changes of mélange thickness coincide with Greenland calving dynamics
Source: Nat Commun. 2025 Jan 10;16:573. doi: 10.1038/s41467-024-55241-7 (PMC11724111; doi:10.1038/s41467-024-55241-7)
Supplement: Supplementary file 1 — Supplementary Information [file 41467_2024_55241_MOESM1_ESM.pdf]

Supplementary Information for Seasonal Changes of Mélange Thickness Coincide With Greenland Calving Dynamics

Yue Meng[1*], Ching-Yao Lai[1], Riley Culberg[2], Michael G. Shahin[3], Leigh A. Stearns[3,4], Justin C. Burton[5], and Kavinda Nissanka[5]

[1]Department of Geophysics, Stanford University, Stanford, CA, USA
[2]Department of Earth and Atmospheric Sciences, Cornell University, Ithaca, NY, USA
[3]Department of Geology, University of Kansas, Lawrence, KS, USA
[4]Department of Earth and Environmental Sciences, University of Pennsylvania, Philadelphia, PA, USA
[5]Department of Physics, Emory University, Atlanta, GA, USA

December 2, 2024

* Corresponding author: olivmeng@stanford.edu

This PDF file contains:
Derivation of the Governing Equations for Ice Mélange
Supplementary Table on the Fitted μ_e in Fig. 4 in the Main Text
Supplementary Table on the List of Glacier Termini in Fig. 8 in the Main Text
Supplementary Figures 1–48

1 Derivation of the governing equations for ice mélange.

We make the following assumptions: (i) the fjord width is a constant; (ii) the mélange is in a three-dimensional state; (iii) the mélange packing density and thickness are uniform across the depth of the mélange, but vary in horizontal directions (x and y); (iv) a viscous constitutive relationship between the mélange deviatoric stress and the strain rate, that is, $\sigma'_{ij} = 2\eta\dot{\epsilon}_{ij}$. As the trace of the deviatoric stress tensor is equal to zero, we assume the mélange flow to be incompressible, that is, $\dot{\epsilon}_{xx} + \dot{\epsilon}_{yy} + \dot{\epsilon}_{zz} = 0$; (v) variations of horizontal velocities across the depth of the mélange are negligible, that is, $\frac{\partial w}{\partial x} \sim \frac{\partial w}{\partial y} \ll \frac{\partial u}{\partial z} \sim \frac{\partial v}{\partial z} \cong 0$, and therefore $\sigma'_{xz} = \sigma'_{yz} = 0$; and (vi) the bottom of the mélange is fully permeable and leaves the skeleton stress-free. Such assumption aligns with the fact that the effective stress always vanishes at the free surface of the solid skeleton in a porous medium [1, 2, 3]. The stress-free boundary condition of the mélange states that $\boldsymbol{\sigma} \cdot \boldsymbol{n} = \mathbf{0}$ at the top and bottom surfaces, where $\mathbf{n}$ is the surface's unit normal vector.

The boundary condition at the top surface states that:

$$
\begin{aligned}
-\sigma_{xx}|_{z_s}\frac{\partial z_s}{\partial x} - \sigma_{xy}|_{z_s}\frac{\partial z_s}{\partial y} + \sigma_{xz}|_{z_s} = 0,\\
-\sigma_{yx}|_{z_s}\frac{\partial z_s}{\partial x} - \sigma_{yy}|_{z_s}\frac{\partial z_s}{\partial y} + \sigma_{yz}|_{z_s} = 0,\\
-\sigma_{zx}|_{z_s}\frac{\partial z_s}{\partial x} - \sigma_{zy}|_{z_s}\frac{\partial z_s}{\partial y} + \sigma_{zz}|_{z_s} = 0,
\end{aligned}
\tag{1}
$$

The boundary condition at the bottom surface states that:

$$
\begin{aligned}
-\sigma_{xx}|_{z_b}\frac{\partial z_b}{\partial x} - \sigma_{xy}|_{z_b}\frac{\partial z_b}{\partial y} + \sigma_{xz}|_{z_b} = 0,\\
-\sigma_{yx}|_{z_b}\frac{\partial z_b}{\partial x} - \sigma_{yy}|_{z_b}\frac{\partial z_b}{\partial y} + \sigma_{yz}|_{z_b} = 0,\\
-\sigma_{zx}|_{z_b}\frac{\partial z_b}{\partial x} - \sigma_{zy}|_{z_b}\frac{\partial z_b}{\partial y} + \sigma_{zz}|_{z_b} = 0,
\end{aligned}
\tag{2}
$$

where z_s, z_b are at the top and bottom surface of the mélange. As we have assumed $\sigma_{xz} = \sigma_{yz} = 0$, we arrive at $\sigma_{zz}|_{z_s} = \sigma_{zz}|_{z_b} = 0$.

Under steady flow conditions, the vertical force balance for ice mélange states that:

$$
\frac{\partial \sigma_{zz}}{\partial z} = \rho_i \phi(x,y) g', \tag{3}
$$

where ρ_i is the density of ice, $\phi(x,y)$ is the packing density of ice mélange that varies along and across the fjord, and g' is the effective acceleration due to gravity. For ice above the waterline, $g' = g$. For ice below the waterline, $g' = (1 - \frac{\rho_w}{\rho_i})g$. Since the vertical stress in ice mélange equals to zero at its top and bottom surface, we arrive at a final expression for σ_{zz}:

$$
\sigma_{zz}(x,y,z) = \begin{cases} \rho_i \phi(x,y) g \left(z - (1 - \frac{\rho_i}{\rho_w}) H(x,y) \right), \text{where } 0 < z < (1 - \frac{\rho_i}{\rho_w}) H(x,y), \\ (\rho_i - \rho_w) \phi(x,y) g \left(z + \frac{\rho_i}{\rho_w} H(x,y) \right), \text{where } -\frac{\rho_i}{\rho_w} H(x,y) < z < 0. \end{cases} \tag{4}
$$

where ρ_w is the density of sea water, and $H(x,y)$ is the mélange thickness that varies along and across the fjord. The equation states that the vertical stress for mélange linearly decreases from zero at the top to $-\rho_i \phi(x,y) g (1 - \frac{\rho_i}{\rho_w}) H(x,y)$ at sea level, and then linearly increases to zero at the bottom.

The horizontal force balances for ice mélange state that:

$$
\begin{aligned}
\frac{\partial \sigma_{xx}}{\partial x} + \frac{\partial \sigma_{xy}}{\partial y} = 0,\\
\frac{\partial \sigma_{yx}}{\partial x} + \frac{\partial \sigma_{yy}}{\partial y} = 0,
\end{aligned}
\tag{5}
$$

Substitute $\sigma_{xx} = -p + \sigma'_{xx} = \sigma_{zz} - \sigma'_{zz} + \sigma'_{xx} = \sigma_{zz} + 2\sigma'_{xx} + \sigma'_{yy}$ and $\sigma_{yy} = -p + \sigma'_{yy} = \sigma_{zz} - \sigma'_{zz} + \sigma'_{yy} = \sigma_{zz} + \sigma'_{xx} + 2\sigma'_{yy}$ into Eqn. (5), we arrive at:

$$
\begin{aligned}
\frac{\partial \sigma_{zz}}{\partial x} + \frac{\partial (2\sigma'_{xx})}{\partial x} + \frac{\partial \sigma'_{yy}}{\partial x} + \frac{\partial \sigma'_{xy}}{\partial y} = 0,\\
\frac{\partial \sigma'_{xy}}{\partial x} + \frac{\partial \sigma_{zz}}{\partial y} + \frac{\partial \sigma'_{xx}}{\partial y} + \frac{\partial (2\sigma'_{yy})}{\partial y} = 0,
\end{aligned}
\tag{6}
$$

From Eqn. (4), we obtain $\frac{\partial \sigma_{zz}}{\partial x}$ and $\frac{\partial \sigma_{zz}}{\partial y}$ as follows:

$$
\begin{aligned}
\frac{\partial \sigma_{zz}}{\partial x} &= -\rho_i g(1-\frac{\rho_i}{\rho_w})\frac{\partial(\phi(x,y)H(x,y))}{\partial x},\\
\frac{\partial \sigma_{zz}}{\partial y} &= -\rho_i g(1-\frac{\rho_i}{\rho_w})\frac{\partial(\phi(x,y)H(x,y))}{\partial y},
\end{aligned}
\tag{7}
$$

Substitute Eqn. (7) into Eqn. (6), we have:

$$
\begin{aligned}
\frac{\partial(2\sigma'_{xx})}{\partial x} + \frac{\partial \sigma'_{yy}}{\partial x} + \frac{\partial \sigma'_{xy}}{\partial y} &= \rho_i g(1-\frac{\rho_i}{\rho_w})\frac{\partial(\phi(x,y)H(x,y))}{\partial x},\\
\frac{\partial \sigma'_{xy}}{\partial x} + \frac{\partial \sigma'_{xx}}{\partial y} + \frac{\partial(2\sigma'_{yy})}{\partial y} &= \rho_i g(1-\frac{\rho_i}{\rho_w})\frac{\partial(\phi(x,y)H(x,y))}{\partial y}.
\end{aligned}
\tag{8}
$$

The stress-free boundary condition of the mélange in Eqn. (1) leads to the expression of $\sigma_{xx}|_{z_s}$ and $\sigma_{yy}|_{z_s}$ as follows:

$$
\begin{aligned}
\sigma_{xx}|_{z_s} &= -p|_{z_s} + \sigma'_{xx}|_{z_s} = \sigma_{zz}|_{z_s} - \sigma'_{zz}|_{z_s} + \sigma'_{xx}|_{z_s} = 2\sigma'_{xx}|_{z_s} + \sigma'_{yy}|_{z_s},\\
\sigma_{yy}|_{z_s} &= -p|_{z_s} + \sigma'_{yy}|_{z_s} = \sigma_{zz}|_{z_s} - \sigma'_{zz}|_{z_s} + \sigma'_{yy}|_{z_s} = \sigma'_{xx}|_{z_s} + 2\sigma'_{yy}|_{z_s},
\end{aligned}
\tag{9}
$$

Therefore, the top boundary condition in Eqn. (1) in horizontal directions (x and y) can be rewritten as follows:

$$
\begin{aligned}
-2\sigma'_{xx}|_{z_s}\frac{\partial z_s}{\partial x} - \sigma'_{yy}|_{z_s}\frac{\partial z_s}{\partial x} - \sigma'_{xy}|_{z_s}\frac{\partial z_s}{\partial y} &= 0,\\
-\sigma'_{xy}|_{z_s}\frac{\partial z_s}{\partial x} - \sigma'_{xx}|_{z_s}\frac{\partial z_s}{\partial y} - 2\sigma'_{yy}|_{z_s}\frac{\partial z_s}{\partial y} &= 0,
\end{aligned}
\tag{10}
$$

Similarly, the bottom boundary condition in the horizontal directions can be written as follows:

$$
\begin{aligned}
-2\sigma'_{xx}|_{z_b}\frac{\partial z_b}{\partial x} - \sigma'_{yy}|_{z_b}\frac{\partial z_b}{\partial x} - \sigma'_{xy}|_{z_b}\frac{\partial z_b}{\partial y} &= 0,\\
-\sigma'_{xy}|_{z_b}\frac{\partial z_b}{\partial x} - \sigma'_{xx}|_{z_b}\frac{\partial z_b}{\partial y} - 2\sigma'_{yy}|_{z_b}\frac{\partial z_b}{\partial y} &= 0,
\end{aligned}
\tag{11}
$$

We integrate Eqn. (8) over the depth of the mélange as follows:

$$
\begin{aligned}
\int_{z_b}^{z_s}\frac{\partial(2\sigma'_{xx})}{\partial x}dz + \int_{z_b}^{z_s}\frac{\partial \sigma'_{yy}}{\partial x}dz + \int_{z_b}^{z_s}\frac{\partial \sigma'_{xy}}{\partial y}dz &= \int_{z_b}^{z_s}\rho_i g(1-\frac{\rho_i}{\rho_w})\frac{\partial(\phi(x,y)H(x,y))}{\partial x}dz,\\
\int_{z_b}^{z_s}\frac{\partial \sigma'_{xy}}{\partial x}dz + \int_{z_b}^{z_s}\frac{\partial \sigma'_{xx}}{\partial y}dz + \int_{z_b}^{z_s}\frac{\partial(2\sigma'_{yy})}{\partial y}dz &= \int_{z_b}^{z_s}\rho_i g(1-\frac{\rho_i}{\rho_w})\frac{\partial(\phi(x,y)H(x,y))}{\partial y}dz,
\end{aligned}
\tag{12}
$$

We then apply Leibniz theorem to Eqn. (12), which becomes:

$$
\begin{aligned}
&\frac{\partial}{\partial x}\int_{z_b}^{z_s}2\sigma'_{xx}dz + \frac{\partial}{\partial x}\int_{z_b}^{z_s}\sigma'_{yy}dz + \frac{\partial}{\partial y}\int_{z_b}^{z_s}\sigma'_{xy}dz - (2\sigma'_{xx}|_{z_s}\frac{\partial z_s}{\partial x} + \sigma'_{yy}|_{z_s}\frac{\partial z_s}{\partial x} + \sigma'_{xy}|_{z_s}\frac{\partial z_s}{\partial y})\\
&\qquad +(2\sigma'_{xx}|_{z_b}\frac{\partial z_b}{\partial x} + \sigma'_{yy}|_{z_b}\frac{\partial z_b}{\partial x} + \sigma'_{xy}|_{z_b}\frac{\partial z_b}{\partial y}) = \rho_i g(1-\frac{\rho_i}{\rho_w})H(x,y)\frac{\partial(\phi(x,y)H(x,y))}{\partial x},\\
&\frac{\partial}{\partial x}\int_{z_b}^{z_s}\sigma'_{xy}dz + \frac{\partial}{\partial y}\int_{z_b}^{z_s}\sigma'_{xx}dz + \frac{\partial}{\partial y}\int_{z_b}^{z_s}2\sigma'_{yy}dz - (\sigma'_{xy}|_{z_s}\frac{\partial z_s}{\partial x} + \sigma'_{xx}|_{z_s}\frac{\partial z_s}{\partial y} + 2\sigma'_{yy}|_{z_s}\frac{\partial z_s}{\partial y})\\
&\qquad +(\sigma'_{xy}|_{z_b}\frac{\partial z_b}{\partial x} + \sigma'_{xx}|_{z_b}\frac{\partial z_b}{\partial y} + 2\sigma'_{yy}|_{z_b}\frac{\partial z_b}{\partial y}) = \rho_i g(1-\frac{\rho_i}{\rho_w})H(x,y)\frac{\partial(\phi(x,y)H(x,y))}{\partial y},
\end{aligned}
\tag{13}
$$

We define the depth-averaged stress $\bar{\sigma}_{ij} = \frac{1}{H}\int_{z_b}^{z_s} \sigma_{ij} dz$, and substitute Eqn. (10), (11) into Eqn. (13). We arrive at the depth-averaged momentum equation in horizontal direction as follows:

$$\frac{\partial}{\partial x}(2H(x,y)\bar{\sigma}'_{xx}) + \frac{\partial}{\partial x}(H(x,y)\bar{\sigma}'_{yy}) + \frac{\partial}{\partial y}(H(x,y)\bar{\sigma}'_{xy}) = \rho_i g(1-\frac{\rho_i}{\rho_w})H(x,y)\frac{\partial(\phi(x,y)H(x,y))}{\partial x},$$
$$\frac{\partial}{\partial x}(H(x,y)\bar{\sigma}'_{xy}) + \frac{\partial}{\partial y}(H(x,y)\bar{\sigma}'_{xx}) + \frac{\partial}{\partial y}(2H(x,y)\bar{\sigma}'_{yy}) = \rho_i g(1-\frac{\rho_i}{\rho_w})H(x,y)\frac{\partial(\phi(x,y)H(x,y))}{\partial y}. \quad (14)$$

We note that this equation resembles the shallow shelf approximation (SSA) describing the flow of ice shelves [4], except that this "granular ice shelf" equation involves the packing density $\phi(x,y)$. The SSA equation for ice mélange (Eqn. (8) or (14)) can be validated by discrete element simulations. We compute the spatial derivatives of the deviatoric stresses in Eqn. (8) from simulations of the thick mélange in straight and rugged fjords (Supplementary Fig. 1). We found that the stress derivative terms in the momentum balance equation in the y direction are one or two order of magnitude smaller than those in the x direction. For the momentum balance in the x direction, the driving stress induced by the mélange thickness gradient is predominantly balanced by the horizontal shear stress. Assuming $\sigma_{xy} = \eta(\frac{\partial u}{\partial y})$, we conduct a simple scaling between the glaciostatic pressure and shear stress terms in Eqn. (8), with the mélange viscosity approximated to be $\eta \sim 2 \times 10^{10}$ Pa·s.

To derive the mélange buttressing force per width on the terminus, we further assume that the packing density, mélange thickness are uniform across the fjord width and only vary in the x direction, and (ii) strain rates and mélange viscosity are uniform across the fjord width and across the depth of the mélange, and only vary in the x direction. Therefore, Eqn. (4) becomes:

$$\sigma_{zz}(x,z) = \begin{cases} \rho_i\phi(x)g\left(z-(1-\frac{\rho_i}{\rho_w})H(x)\right), \text{where } 0 < z < (1-\frac{\rho_i}{\rho_w})H(x), \\ (\rho_i-\rho_w)\phi(x)g\left(z+\frac{\rho_i}{\rho_w}H(x)\right), \text{where } -\frac{\rho_i}{\rho_w}H(x) < z < 0. \end{cases} \quad (15)$$

which leads to the expression of the width-averaged buttressing force as follows:

$$\begin{aligned}\frac{F}{W} &= \left(\int_{z_b}^{z_s} -\sigma_{xx}(x,z)dz\right)|_{x=0} = \left(\int_{z_b}^{z_s}(p(x,z)-\sigma'_{xx}(x))dz\right)|_{x=0} \\ &= \left(\int_{z_b}^{z_s}(\sigma'_{zz}(x)-\sigma_{zz}(x,z)-\sigma'_{xx}(x))dz\right)|_{x=0} = \left(\int_{z_b}^{z_s}(-\sigma_{zz}(x,z)-2\sigma'_{xx}(x)-\sigma'_{yy}(x))dz\right)|_{x=0} \\ &= \frac{1}{2}\rho_i(1-\frac{\rho_i}{\rho_w})g\phi_0 H_0^2 - 4H_0(\eta\frac{\partial u}{\partial x})|_{x=0} - 2H_0(\eta\frac{\partial v}{\partial y}))|_{x=0}.\end{aligned} \quad (16)$$

where η, $\frac{\partial u}{\partial x}$ and $\frac{\partial v}{\partial y}$ are the viscosity and strain rates of the mélange, respectively. ϕ_0, H_0 are the mélange packing density and thickness at the terminus, respectively.

Finally, we derive the expression for the mélange thickness profile, $H(x)$. Eqn. (14) can be reorganized as follows:

$$\frac{\partial}{\partial y}(H(x)\bar{\sigma}'_{xy}) = -\frac{\partial}{\partial x}(H(x)\bar{\sigma}_{zz}) - \frac{\partial}{\partial x}(2H(x)\bar{\sigma}'_{xx}) - \frac{\partial}{\partial x}(H(x)\bar{\sigma}'_{yy}) \quad (17)$$

Because we assume mélange thickness and stresses do not vary with y, we can integrate Eqn. (17) over the y direction as:

$$(H(x)\bar{\sigma}'_{xy})|_{y=W} - (H(x)\bar{\sigma}'_{xy})|_{y=0} = W(-\frac{\partial}{\partial x}(H(x)\bar{\sigma}_{zz}) - \frac{\partial}{\partial x}(2H(x)\bar{\sigma}'_{xx}) - \frac{\partial}{\partial x}(H(x)\bar{\sigma}'_{yy})) \quad (18)$$

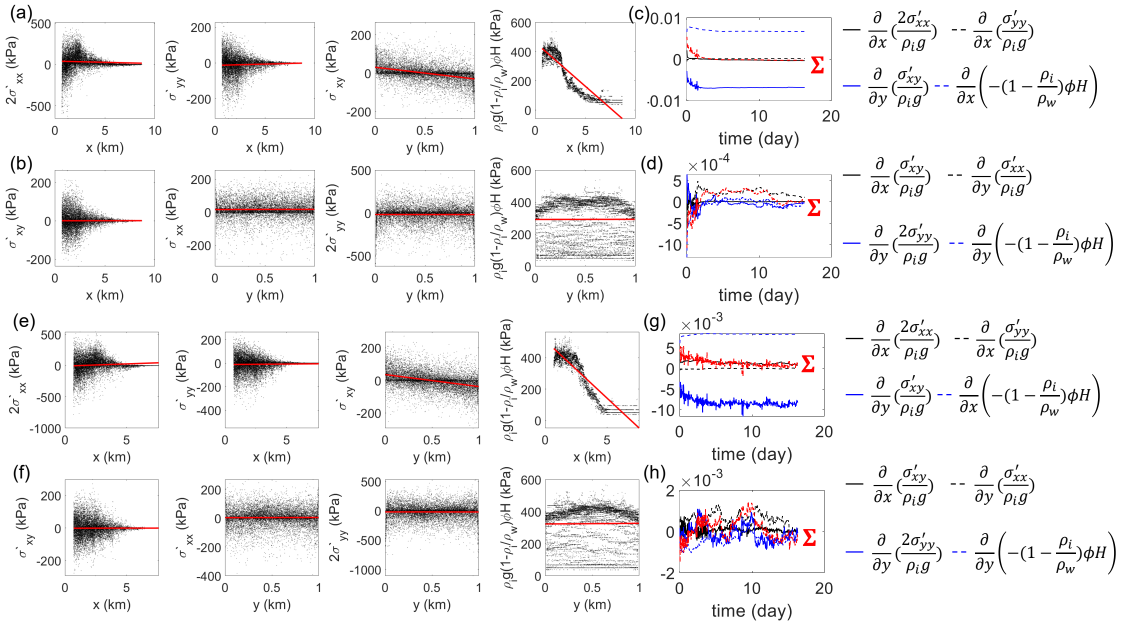

Supplementary Figure 1: Validating the mélange SSA equations (Eqn. (8)) using the discrete element model for a thick mélange ($H_{\text{ini}} = 378$ m) in the straight ((a)-(d)) and rugged ((e)-(h)) fjords. (a) and (b): The spatial distribution of the deviatoric stresses from the x and y direction momentum balances in Eqn. (8) at $t = 16$ days. Black dots represent stresses for each particle in the mélange, and the red lines are the linear fits to the data to find the spatial derivatives. (c) and (d): The temporal evolution of the spatial derivatives of the deviatoric stresses from the x and y direction momentum balances in Eqn. (8), the summation of which are close to zero as indicated by the red dashed lines. (e)-(h) follow legends for (a)-(d).

We use Coulomb friction law to calculate the shear stress at the fjord walls as follows:

$$\begin{aligned} \bar{\sigma}'_{xy}|_{y=W} &= \mu_e \bar{\sigma}_{yy}, \\ \bar{\sigma}'_{xy}|_{y=0} &= -\mu_e \bar{\sigma}_{yy}, \end{aligned} \tag{19}$$

where μ_e is the effective coefficient of friction between the mélange and the fjord wall, which depends on the material friction coefficient and the geometry of the fjord walls, i.e., wall roughness [5, 6, 7]. As $\bar{\sigma}_{yy} = \bar{\sigma}_{zz} + \bar{\sigma}'_{xx} + 2\bar{\sigma}'_{yy}$, Eqn. (18) becomes

$$\frac{2H(x)\mu_e}{W}(\bar{\sigma}_{zz} + \bar{\sigma}'_{xx} + 2\bar{\sigma}'_{yy}) = -\frac{\partial}{\partial x}(H(x)\bar{\sigma}_{zz}) - \frac{\partial}{\partial x}(2H(x)\bar{\sigma}'_{xx}) - \frac{\partial}{\partial x}(H(x)\bar{\sigma}'_{yy}) \tag{20}$$

Following the scaling analysis in the main text (Eqn. 4 in section 1.3), we can reasonably assume that $\bar{\sigma}'_{xx}, \bar{\sigma}'_{yy} \ll \bar{\sigma}_{zz}$, where the depth-averaged vertical stress is $\bar{\sigma}_{zz} = \frac{1}{2}\rho_i g \phi(x)(1 - \frac{\rho_i}{\rho_w})H(x)$. We further assume that the mélange packing density remains a constant along fjords. Therefore, Eqn. (20) becomes

$$\frac{\partial H}{\partial x} + \frac{\mu_e}{W}H(x) = 0 \tag{21}$$

which gives

$$H(x) = Ce^{-\frac{\mu_e}{W}x} \tag{22}$$

where C is a constant that needs to be constrained by a boundary condition of the thickness profile. The mélange thickness exponentially decays with the distance from terminus. However, Eqn. (22) only holds when mélange can be considered as a three-dimensional material. When the mélange thickness decays to a monolayer of icebergs, its thickness is dictated by the iceberg size distribution, instead of stress balances that give rise to Eqn. (22). We identify the mélange length, L, where the mélange thickness decays to a threshold value, H_L, below which the mélange is considered to be a two-dimensional material. Using the boundary condition, $H(x = L) = H_L$, we arrive at the final expression for the mélange thickness profile:

$$H(x) = H_L e^{\frac{\mu_e L}{W}(1-\frac{x}{L})}, x \in [0, L] \tag{23}$$

By defining the dimensionless distance, $\tilde{x} = \frac{\mu_e x}{W}$, and the dimensionless thickness, $\tilde{H} = \frac{H(x)}{H_L e^{\frac{\mu_e L}{W}}}$, we arrive at the dimensionless form of the mélange thickness profile:

$$\tilde{H}(\tilde{x}) = e^{-\tilde{x}}, \tilde{x} \in [0, \frac{\mu_e L}{W}]. \tag{24}$$

2 Supplementary Tables.

Supplementary Table 1: The fitted fjord effective coefficient of friction (μ_e) from mélange thickness profile in Fig. 4 in the main text. The uncertainties come from the mélange thickness threshold, $H_L = 36 \pm 10$ m.

Glacier name	ArcticDEM date	μ_e (ArcticDEM)	μ_e (Discrete element model)
Kangerlussuaq	2020/05/05	$0.64^{+0.09}_{-0.06}$	$0.77^{+0.11}_{-0.06}$
Kangerlussuaq	2018/04/12	$0.39^{+0.05}_{-0.04}$	$0.48^{+0.07}_{-0.04}$
Helheim	2014/08/17	$0.25^{+0.03}_{-0.03}$	$0.39^{+0.05}_{-0.05}$

Supplementary Table 2: The list of glacier termini to compose the Greenland mélange buttressing map in Fig. 8 in the main text

ID	Glacier name	H_i (m) *1	No. DEM	$H_0^{\min}$ (m) *2	$H_0^{\max}$ (m) *2	$F^{\min}/W$ (N/m) *3	$F^{\max}/W$ (N/m) *3
1	Tuttulikassaap Sermia (Hayes Glacier)	491 ± 37	4	N/A	145^{+36}_{-44} (Mar)	N/A	$9.76^{+4.76}_{-5.44} \times 10^6$
2	Hayes Glacier 2	513 ± 22	3	24^{+15}_{-13} (Mar)	49^{+19}_{-19} (Apr)	$2.74^{+4.11}_{-2.21} \times 10^5$	$1.10^{+0.94}_{-0.73} \times 10^6$
3	Hayes Glacier SS	517 ± 43	4	30^{+16}_{-14} (Aug)	80^{+25}_{-27} (Mar)	$4.05^{+5.11}_{-3.08} \times 10^5$	$2.96^{+1.88}_{-1.79} \times 10^6$
4	Nunatakassaap Sermia (Alison Glacier)	665 ± 35	9	10^{+12}_{-9} (Jul)	156^{+37}_{-47} (Apr)	$4.23^{+17.20}_{-4.23} \times 10^4$	$1.12^{+0.53}_{-0.62} \times 10^7$
5	Sermeq N (Upernavik Isstrøm N)	600 ± 28	3	12^{+13}_{-10} (Aug)	148^{+36}_{-45} (Apr)	$6.86^{+21.0}_{-6.66} \times 10^4$	$1.01^{+0.49}_{-0.56} \times 10^7$
6	Kangilliup Sermia (Rink Isbræ)	437 ± 9	3	N/A	79^{+24}_{-27} (May)	N/A	$2.90^{+1.85}_{-1.76} \times 10^6$
7	Kangilleq	259 ± 46	1	24^{+15}_{-13} (Jun)	N/A	$2.74^{+4.11}_{-2.21} \times 10^5$	N/A
8	Sermeq Kujalleq (Jakobshavn Isbræ)	892 ± 33	8	24^{+15}_{-13} (May)	233^{+51}_{-67} (Mar)	$2.74^{+4.11}_{-2.21} \times 10^5$	$2.51^{+1.06}_{-1.34} \times 10^7$
9	Unnamed Deception	446 ± 39	6	40^{+18}_{-17} (Aug)	170^{+40}_{-51} (Mar)	$7.41^{+7.34}_{-5.19} \times 10^5$	$1.33^{+0.62}_{-0.73} \times 10^7$
10	Unnamed Uunartit Islands	299 ± 16	3	N/A	137^{+34}_{-42} (Apr)	N/A	$8.63^{+4.30}_{-4.84} \times 10^6$
11	Helheim Glacier	692 ± 54	2	45^{+19}_{-19} (Jun)	141^{+25}_{-37} (Nov)	$9.46^{+8.57}_{-6.43} \times 10^5$	$9.18^{+4.53}_{-5.13} \times 10^6$
12	Ikertivaq S	131 ± 55	2	9^{+12}_{-9} (Aug)	N/A	$3.50^{+16.0}_{-3.50} \times 10^4$	N/A
13	Koge Bugt C	433 ± 42	2	43^{+18}_{-18} (May)	114^{+30}_{-36} (Jul)	$8.40^{+7.95}_{-5.80} \times 10^5$	$6.01^{+3.22}_{-3.44} \times 10^6$
14	Koge Bugt S	452 ± 34	4	26^{+15}_{-14} (Jul)	118^{+31}_{-37} (Apr)	$3.15^{+4.44}_{-2.48} \times 10^5$	$6.47^{+3.42}_{-3.69} \times 10^6$
15	Gråulv	439 ± 62	2	24^{+15}_{-13} (Oct)	59^{+21}_{-22} (Mar)	$2.74^{+4.11}_{-2.21} \times 10^5$	$1.62^{+1.23}_{-1.04} \times 10^6$
16	Gyldenlove	415 ± 27	5	35^{+17}_{-16} (Oct)	112^{+30}_{-36} (Mar)	$5.60^{+6.19}_{-4.07} \times 10^5$	$5.82^{+3.15}_{-3.35} \times 10^6$
17	A.P. Bernstorff Glacier	474 ± 68	4	38^{+17}_{-17} (Oct)	132^{+33}_{-41} (Apr)	$6.78^{+6.95}_{-4.80} \times 10^5$	$7.98^{+4.04}_{-4.50} \times 10^6$
18	Storebjørn	130 ± 24	4	2^{+11}_{-2} (Jun)	63^{+22}_{-23} (Apr)	$1.40^{+71.7}_{-1.40} \times 10^3$	$1.81^{+1.33}_{-1.15} \times 10^6$
19	Mogens Heinesen S	313 ± 74	1	62^{+21}_{-23} (Aug)	N/A	$1.76^{+1.30}_{-1.12} \times 10^6$	N/A
20	Nansen Glacier	353 ± 21	1	53^{+20}_{-21} (Apr)	N/A	$1.30^{+1.06}_{-0.85} \times 10^6$	N/A
21	Kjer Glacier	305 ± 36	3	11^{+13}_{-10} (Oct)	107^{+29}_{-34} (Apr)	$5.91^{+19.7}_{-5.81} \times 10^4$	$5.29^{+2.92}_{-3.06} \times 10^6$
22	Sermeq (Upernavik Isstrøm)	627 ± 44	7	18^{+14}_{-12} (Aug)	231^{+50}_{-66} (Mar)	$1.54^{+3.05}_{-1.35} \times 10^5$	$2.46^{+1.04}_{-1.31} \times 10^7$
23	Apuseerajik (Fenris Glacier)	597 ± 54	2	21^{+14}_{-12} (Jul)	102^{+28}_{-33} (Oct)	$2.02^{+3.49}_{-1.70} \times 10^5$	$4.79^{+2.70}_{-2.79} \times 10^6$
24	Nigertiip Apusiia (Midgård Glacier)	212 ± 32	3	26^{+15}_{-14} (Jun)	86^{+26}_{-29} (Apr)	$3.15^{+4.44}_{-2.48} \times 10^5$	$3.43^{+2.10}_{-2.05} \times 10^6$
25	Krusse Fj.	130 ± 11	3	1^{+11}_{-1} (Oct)	100^{+28}_{-33} (Apr)	$0.04^{+6.16}_{-0.04} \times 10^4$	$4.63^{+2.63}_{-2.70} \times 10^6$
26	Sermeq Kujalleq	526 ± 111	1	18^{+14}_{-12} (May)	N/A	$1.54^{+3.05}_{-1.35} \times 10^5$	N/A
27	Sermeq Kujalleq (Store Glacier)	608 ± 62	1	51^{+19}_{-20} (May)	N/A	$1.18^{+0.99}_{-0.78} \times 10^6$	N/A
28	Nuussuup Sermia (Kong Oscar Glacier)	685 ± 24	4	N/A	120^{+31}_{-38} (May)	N/A	$6.66^{+3.50}_{-3.80} \times 10^6$
29	Zachariae Isstrøm	467 ± 46	1	24^{+15}_{-13} (Jul)	N/A	$2.74^{+4.11}_{-2.21} \times 10^5$	N/A
30	Daugaard-Jensen Glacier	459 ± 91	1	N/A	61^{+21}_{-23} (May)	N/A	$1.71^{+1.28}_{-1.09} \times 10^6$
31	Kangerlussuaq Glacier	816 ± 48	8	75^{+24}_{-26} (Aug)	241^{+52}_{-69} (Apr)	$2.59^{+1.71}_{-1.58} \times 10^6$	$2.69^{+1.12}_{-1.43} \times 10^7$
32	K.J.V. Steenstrup Nordre Bræ	282 ± 30	3	7^{+12}_{-7} (Jul)	N/A	$2.24^{+13.7}_{-2.24} \times 10^4$	N/A

*1 The averaged ice thickness at the glacier terminus and corresponding uncertainties are obtained from BedMachine [8].

*2 $H_0^{\min}$ is the thinnest mélange observed during terminus retreating periods. $H_0^{\max}$ is the thickest mélange observed during terminus advancing periods.

*3 We calculate the mélange buttressing force, $F^{\min}/W$ and $F^{\max}/W$, with mélange thickness equals to $H_0^{\min}$ and $H_0^{\max}$, respectively.

3 Supplementary Figures.

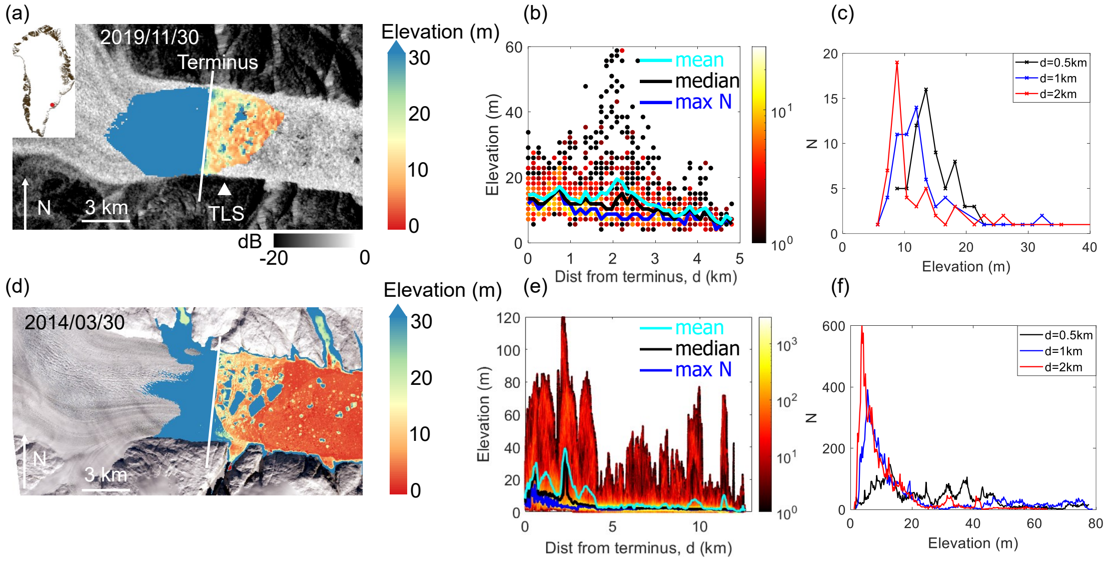

Supplementary Figure 2: Helheim Glacier and ice mélange. (a) TLS-measured elevation map after accounting for local differences between the ellipsoid and geoid with tidal corrections [9], overlain on a Sentinel-1 HV image (both acquired on 30 Nov 2019). The white line across the fjord indicates the glacier front location. The white triangle indicates the TLS location. The upper left inset shows the location of Helheim Glacier in Greenland. The image is in polar stereographic projection (EPSG: 3413). (b) Surface elevation profile for the mélange displayed as a density plot; the colour bar denotes the number of data points that have the same elevation and distance from terminus values. For any specific distance from terminus, we find the elevation value that has the maximum number of data points. The solid blue line connects these elevation values along the distance from terminus as the representative mélange elevation profile. We also calculate the median and mean elevation values for each specific distance from terminus, and connect them by solid black and cyan lines, respectively. (c) The number of data points against the surface elevation value at distance from terminus of 0.5 km (black line), 1 km (blue line), and 2 km (red line). Due to the long-tail distribution of the mélange elevation, the median or mean elevation value approach in (b) tends to incorporate large icebergs into the elevation profile, failing to represent the elevation piled up from small icebergs and sea ice. (d) ArcticDEM measured elevation map after accounting for local differences between the ellipsoid and geoid with tidal corrections [9], overlain on a Landsat 8 image (acquired on 30 Mar 2014). The image is in polar stereographic projection (EPSG: 3413). (e) and (f) follow captions of (b) and (c).

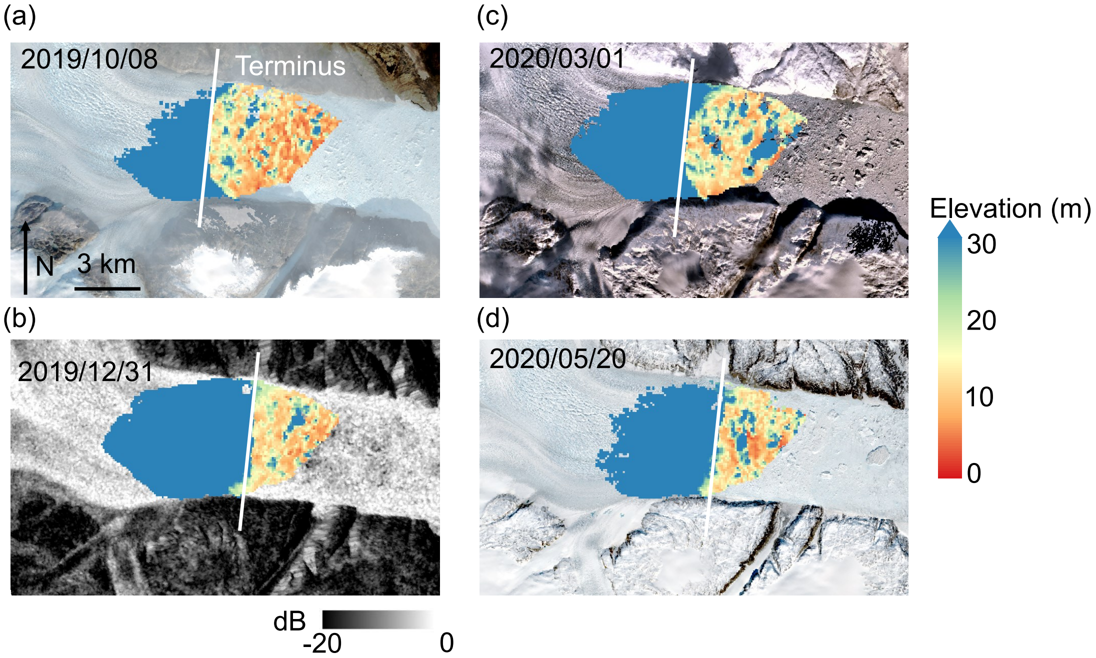

Supplementary Figure 3: The TLS-measured mélange surface elevation map at Helheim Glacier during two episodes of calving cessation, from (a, b) 8 Oct 2019 – 31 Dec 2019, and (c, d) 1 Mar 2020 – 20 May 2020. White lines indicate positions of the terminus. Dates on the images show the acquisition dates for TLS data. TLS scans are overlain on Sentinel-1 and 2 images acquired around the same dates. Images are in polar stereographic projection (EPSG: 3413).

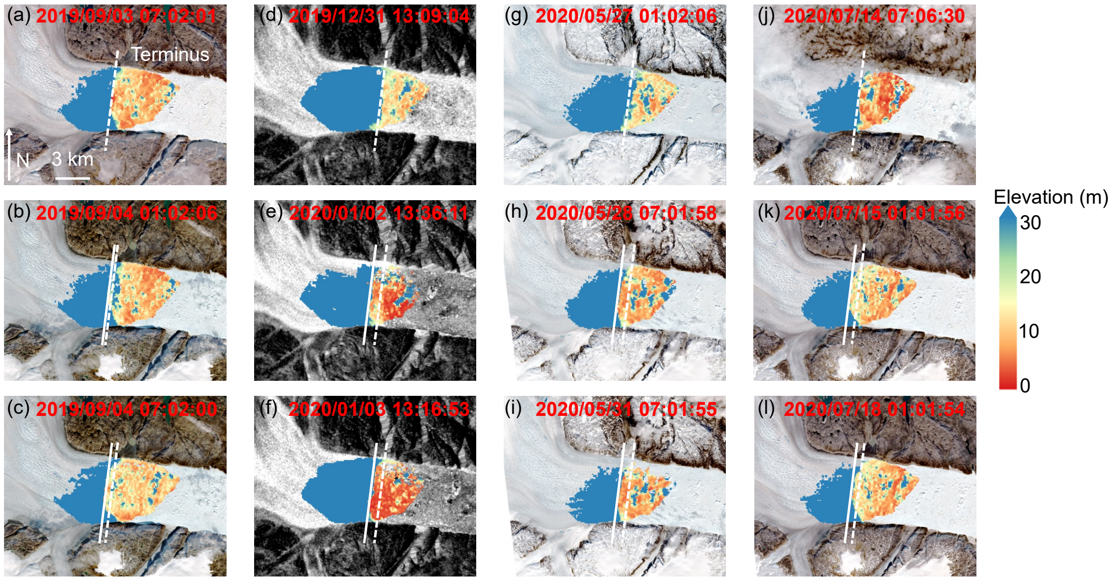

Supplementary Figure 4: The TLS-measured mélange surface elevation map at Helheim Glacier during four major calving events, from (a)-(c) 3 Sep 2019 – 4 Sep 2019, (d)-(f) 31 Dec 2019 – 3 Jan 2020, (g)-(i) 27 May 2020 – 31 May 2020, and (j)-(l) 14 Jul 2020 – 18 Jul 2020. Dashed white lines indicate positions of the terminus before calving. Solid white lines indicate positions of the terminus after calving. Dates on the images show the acquisition dates for TLS data and times are in UTC. TLS scans are overlain on Sentinel-1 and 2 images acquired around the same dates. Images are in polar stereographic projection (EPSG: 3413).

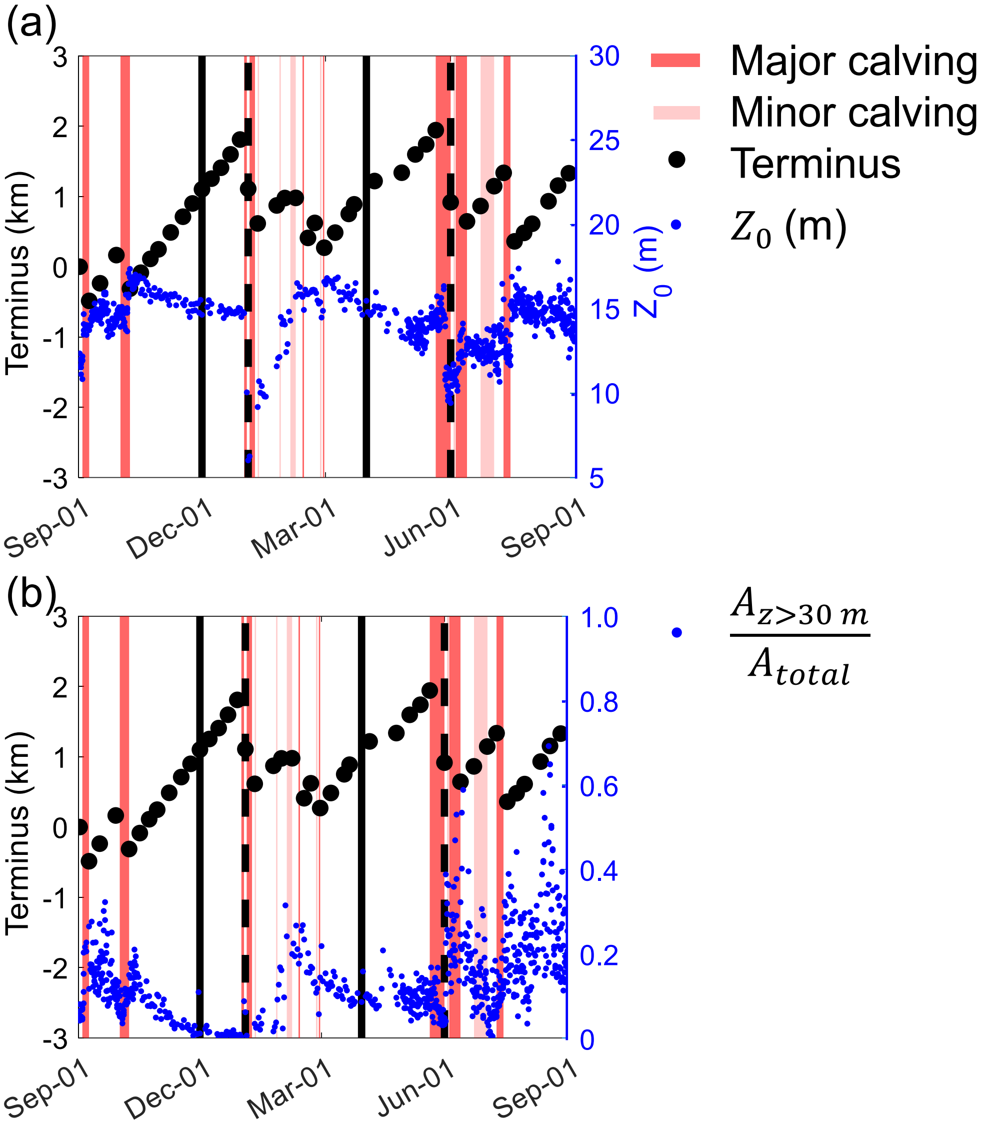

Supplementary Figure 5: Helheim Glacier and ice mélange. (a) Terminus position relative to 1 Sep 2019, where the positive sign indicates terminus advance. Blue dots denote the averaged mélange elevation within 1 km of the terminus, Z_0. Calving events are inferred from TLS and satellite images. Due to limited temporal sampling of the data, we are not able to determine the exact time of each calving event. Instead, we mark the time period during which a calving event occurs by a red-shade rectangle. Four vertical black lines mark the dates for the TLS-measured elevation data presented in Fig. 1(c)-(f) in the main text, which corresponds to 30 Nov 2019, 3 Jan 2020, 30 Mar 2020, and 31 May 2020, respectively. Solid black lines mark the dates with terminus advances, and dashed black lines mark the dates with terminus retreats. (b) follows legends in (a), except blue dots here denote the ratio between the TLS-measured area of mélange with a freeboard height above 30 m ($A_{z>30\ m}$) and the total area of mélange (A_{total}) within 1 km of the terminus.

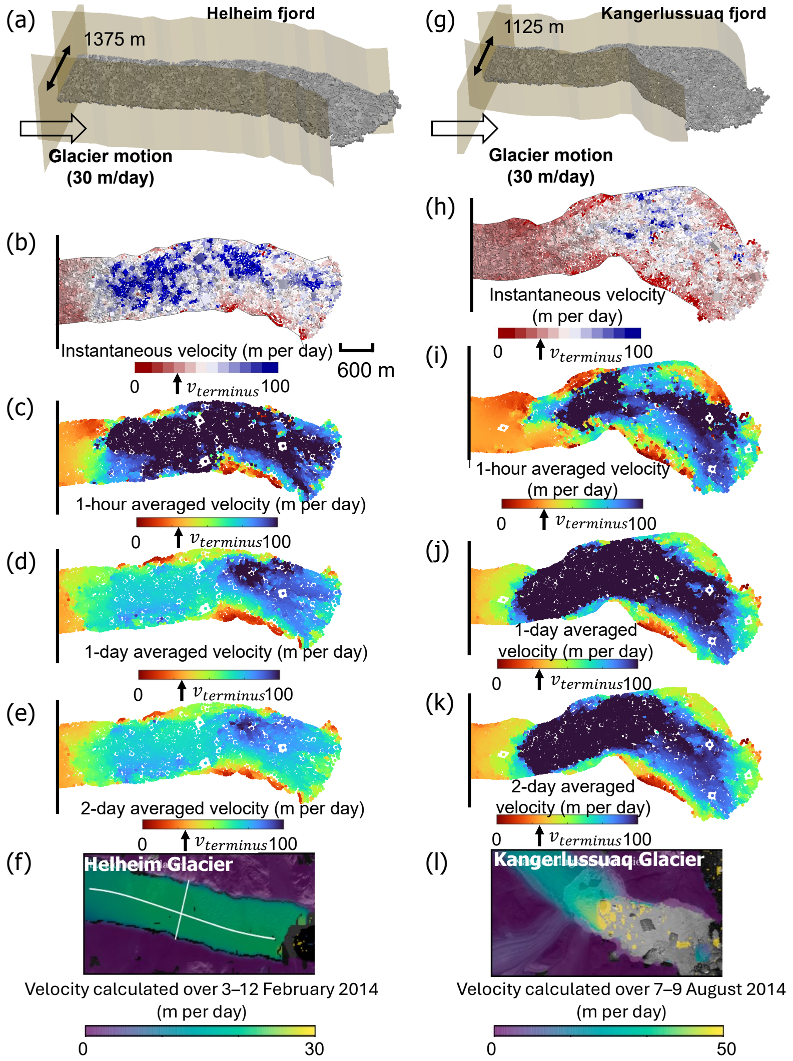

Supplementary Figure 6: The three-dimensional discrete element model for mélange composed of cubic icebergs with a power-law size distribution and confined within real fjord geometries. (a)-(e) For Helheim glacier, (g)-(k) for Kangerlussuaq glacier. The real fjord geometry is scaled down by four times for the discrete element model. (a) The side view for iceberg positions after 16 days into simulations with steady terminus advance and no calving. The glacier terminus moves at a constant velocity, $V_{\mathrm{ter}} = 30$ m/day. (b) The top view of the instantaneous velocity of iceberg element indicated by filled colour. We also calculate the time averaged velocity of each iceberg element by dividing the iceberg's displacement over one hour, one day, and two days of terminus motion by the corresponding time intervals. We indicate the 1-hour averaged (c), 1-day averaged (d), and 2-day averaged (e) velocity field by filled colour. (g)-(k) follow captions of (a)-(e). See supplementary videos for the full temporal evolution of the mélange behaviors. Remote observations of uniform (f) and extensional flow (l) regimes of ice mélange adapted with permission from [10]. Velocities were calculated over 3–12 February 2014 at Helheim Glacier (f), and 7–9 August 2014 at Kangerlussuaq Glacier (l). Gray regions indicate where image cross-correlation results were discarded due to low signal-to-noise ratios.

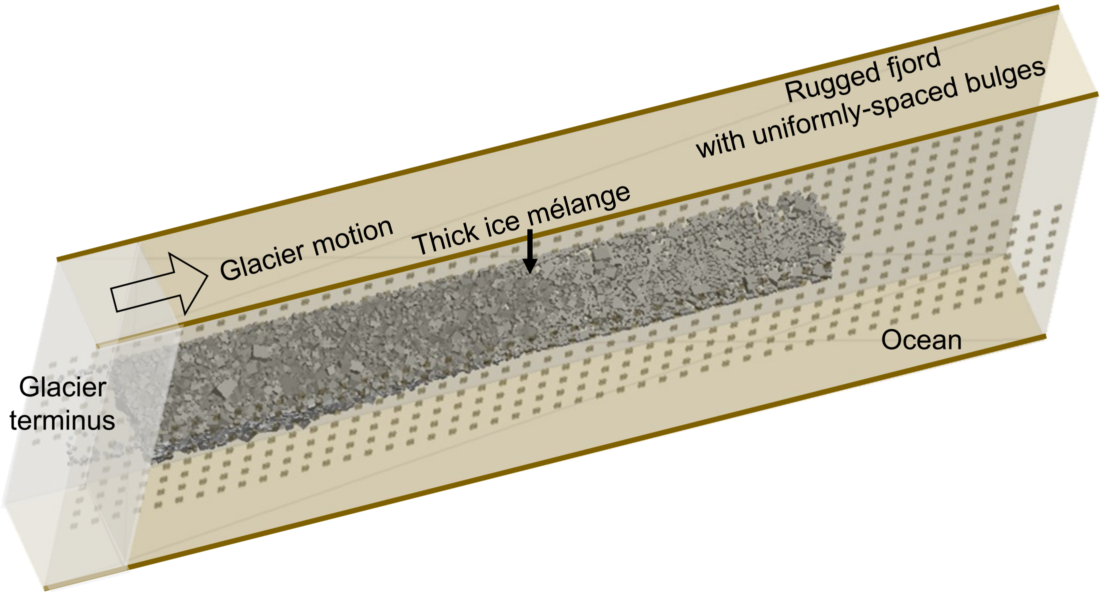

Supplementary Figure 7: A perspective view of the three-dimensional discrete element model for the thick mélange confined within the rugged fjord wall configuration (the simulation shown in Fig. 5 (h)(j) in the main text).

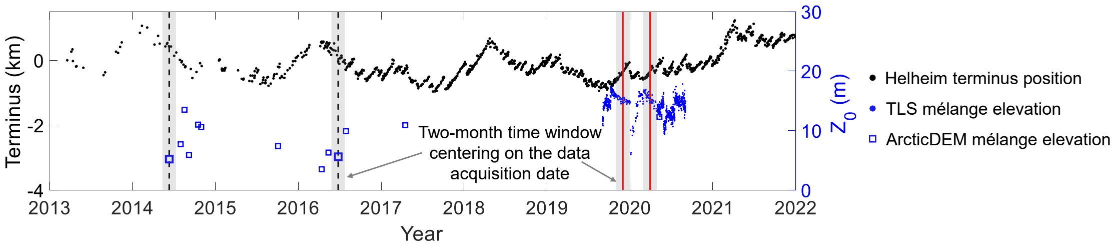

Supplementary Figure 8: A time series of terminus position [11] and observed mélange freeboard heights (Z_0) at the terminus of Helheim Glacier. Black dots denote the terminus position where the positive sign indicates terminus advance. Blue dots and squares denote the mélange elevation observed at the terminus, Z_0, from TLS and ArcticDEM strips, respectively. The solid red (or dashed black) line marks the data acquisition date when the terminus consistently advances (or retreats) within the two-month time window centering on that date, indicating that the DEM potentially represents mélange with a strong (or weak) buttressing force. We exclude DEMs when the terminus behavior alternates between advancing and retreating within the two-month time window, because the relationship between mélange and calving dynamics is ambiguous in this case. We present only the filtered DEM data (i.e., four data points at Helheim Glacier) in Figure 8 in the main text.

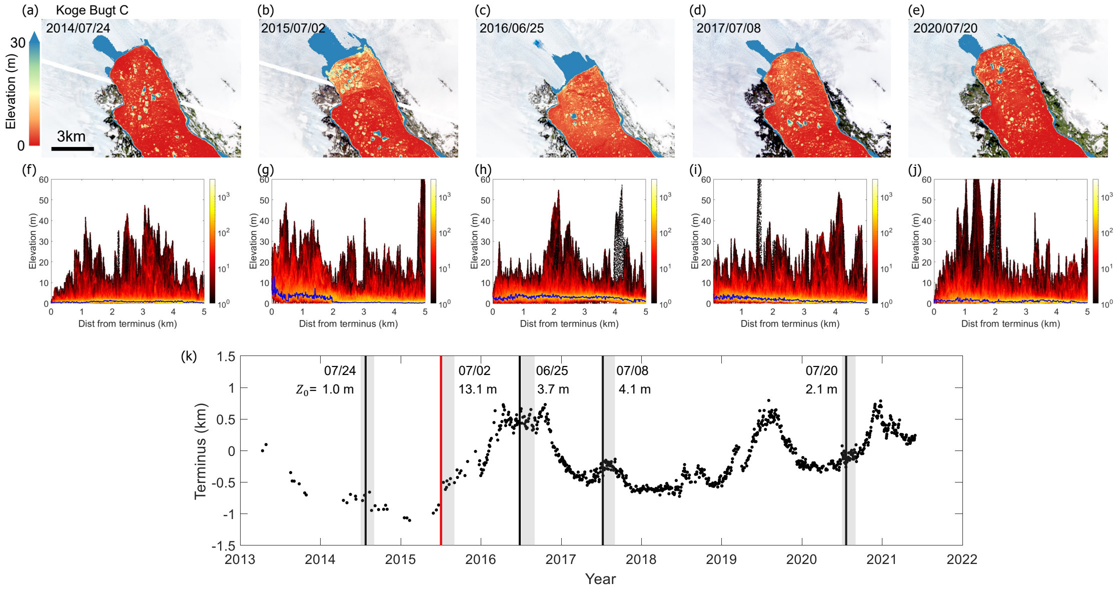

Supplementary Figure 9: (a)-(e) The mélange elevation map above mean sea level at Koge Bugt C Glacier from ArcticDEM observations, acquired in June and July from 2014–2020, overlain on the satellite image acquired around the same date. The image is in polar stereographic projection (EPSG: 3413). The date of ArcticDEM acquisition is shown on the upper left corner. (f)-(j) The surface elevation profile for the mélange presented in (a)–(e) displayed as a density plot. The solid blue line is the representative mélange elevation profile as a function of distance from terminus. (k) A time series of terminus position [11], ArcticDEM acquisition dates and observed mélange freeboard heights (Z_0) at the terminus of Koge Bugt C Glacier. The solid red line marks the ArcticDEM acquisition date when an unusually thick mélange is observed at the terminus in July with the terminus advancing from May to October in 2015. The solid black lines mark ArcticDEM acquisition dates around the same time (late June to July) in different years (2014, 2016, 2017, 2020) when thin mélange is observed at the terminus with the terminus retreating or remaining a plateau from July to September (the shaded gray regions).

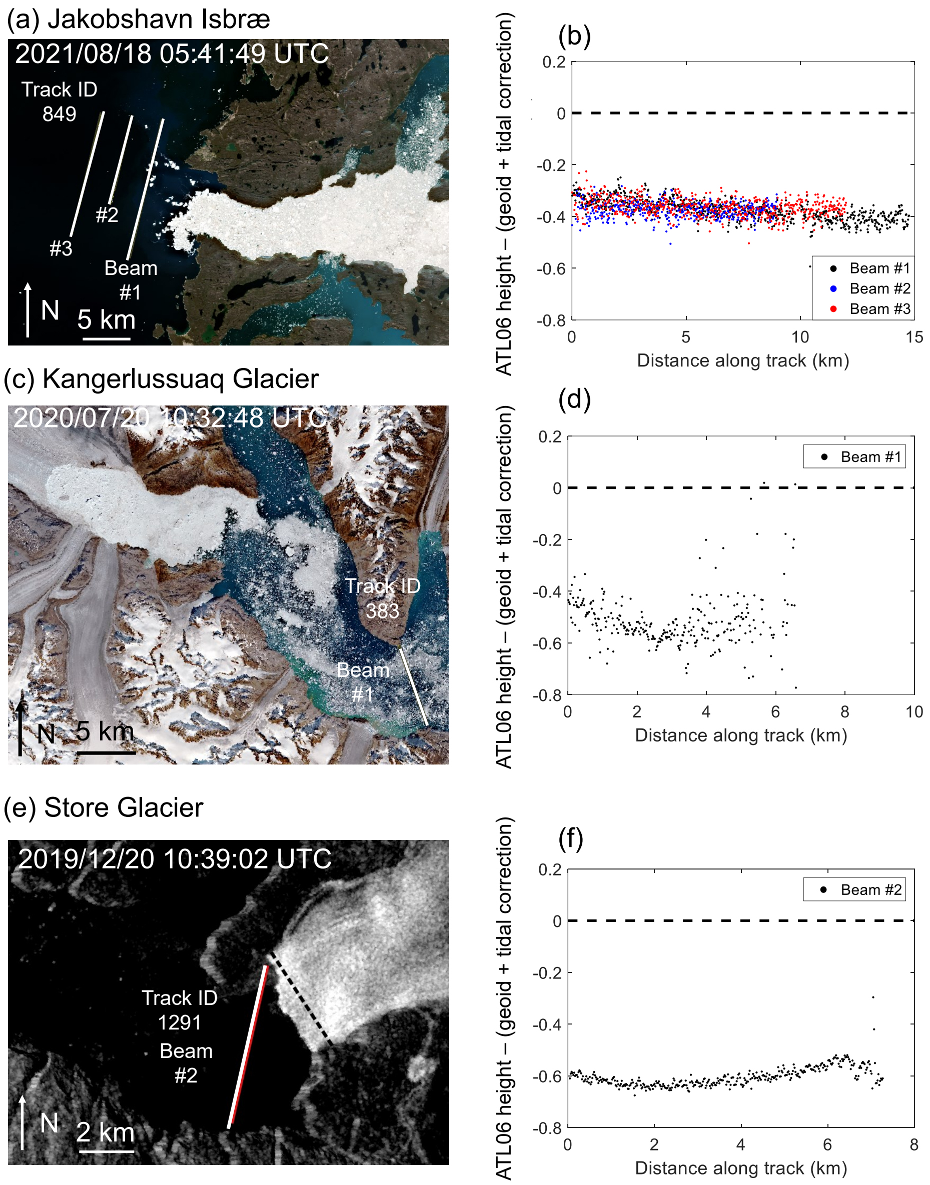

Supplementary Figure 10: The mélange elevation data uncertainty by computing the difference between ICESat-2 ATL06 measurements over the ocean and the geoid [12] with tidal corrections [9], at Jakobshavn Isbræ (a)(b), Kangerlussuaq Glacier (c)(d), and Store Glacier (e)(f). (a) The Sentinel-2 image for Jakobshavn Isbræ on 17 Aug 2021. In (a)(c)(e), the white lines indicate ICESat-2 tracks along which ATL06 surface elevation data was acquired. The date and time on the image shows the acquisition time for ICESat-2 data, which is around the same date of the presented satellite image. Images are in polar stereographic projection (EPSG: 3413). The ICESat-2 track and beam IDs are presented in white texts. (c) The Sentinel-2 image for Kangerlussuaq Glacier on 19 Jul 2020. (e) The Sentinel-1 HV image for Store Glacier on 19 Dec 2019. (b)(d)(f) The difference between ATL06 measurements over the ocean and the geoid [12] with tidal corrections [9] as a function of the distance along the ICESat-2 track in (a)(c)(e).

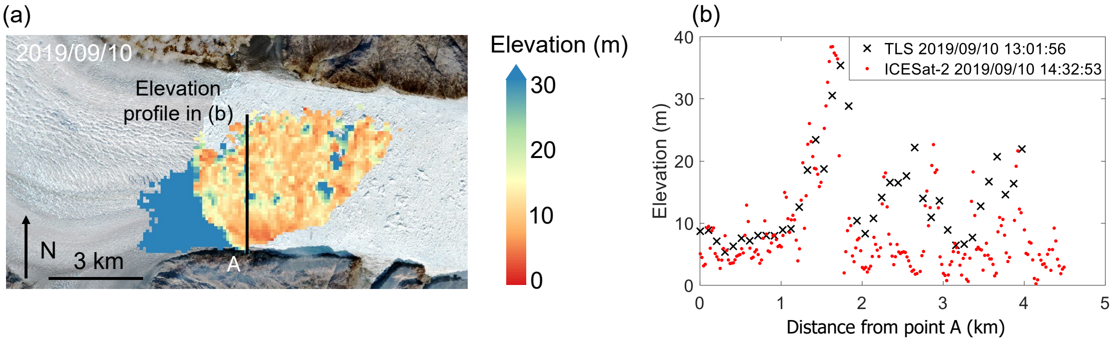

Supplementary Figure 11: A comparison of mélange surface elevation from TLS and ICESat-2 at Helheim Glacier. (a) TLS-measured elevation map after accounting for local differences between the ellipsoid and geoid [12] with tidal corrections [9], overlain on a Sentinel-2 image (both acquired on 10 Sep 2019). The black line across the fjord indicates the ICESat-2 track along which surface elevation data is acquired. Images are in polar stereographic projection (EPSG: 3413). (b) Surface elevation profiles extracted along the black line in (a). Black cross markers indicate data acquired from TLS on 10 Sep 2019, 13:01:56 UTC, and red dot markers indicate data acquired from ICESat-2 on 10 Sep 2019, 14:32:53 UTC. The horizontal axis shows the distance from point A in (a).

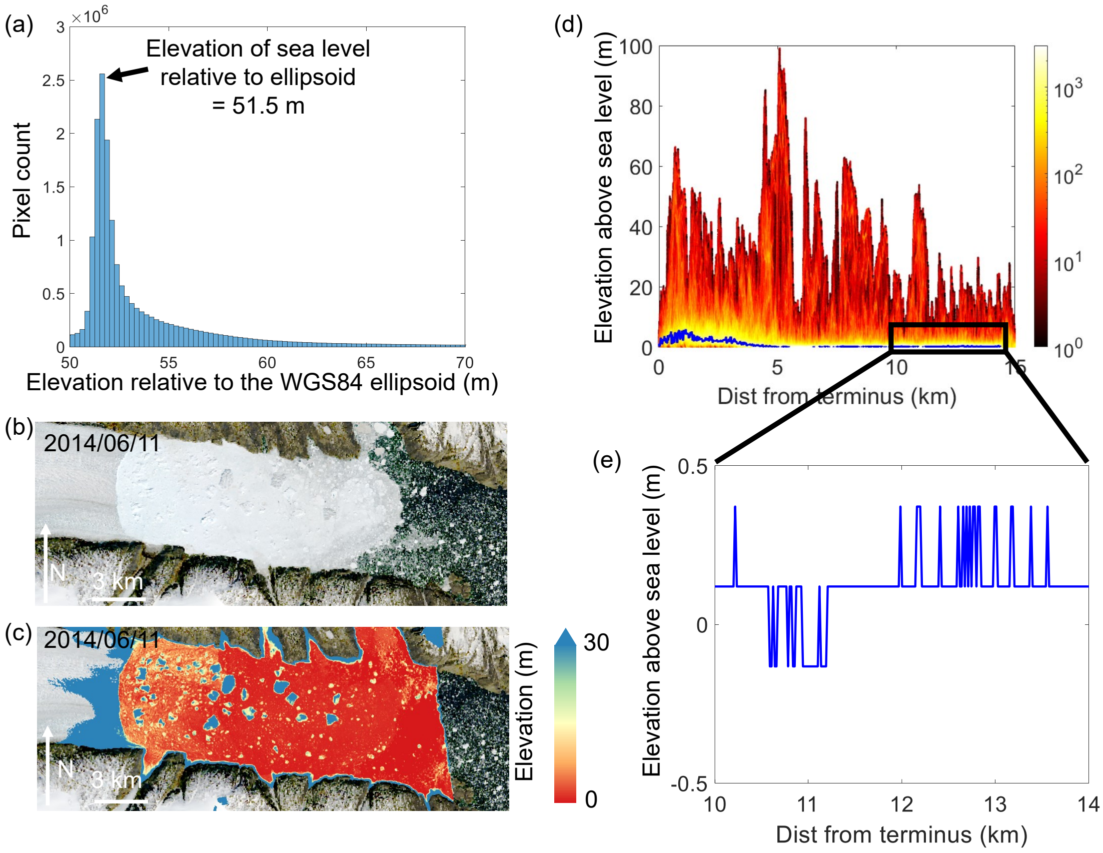

Supplementary Figure 12: The calculation of sea level for an ArcticDEM strip that covers mélange and ocean areas. (a) Example of a histogram of elevation pixel count in an ArcticDEM image at Helheim Glacier acquired on 11 Jun 2014. The elevation with the highest pixel count is automatically selected as the sea level for that scene. In this example sea level would be 51.5 m relative to the WGS84 ellipsoid. (b) The Landsat 8 image for Helheim Glacier on 11 Jun 2014 in polar stereographic projection (EPSG: 3413). (c) The mélange elevation above sea level from the ArcticDEM strip acquired on 11 Jun 2014 overlain on the satellite image in (b). The ArcticDEM strip is registered to the detected sea level in (a) by subtracting 51.5 m from the elevation values relative to the WGS84 ellipsoid. (d) The surface elevation profile for the mélange displayed as a density plot. The solid blue line is the representative mélange elevation profile. (e) A zoom-in view of the representative mélange elevation profile with the distance from terminus varying from 10 km to 14 km. The region is mostly ocean area (i.e. 0 m above sea level) with the detected elevation above sea level varying from -0.13 m to 0.37 m.

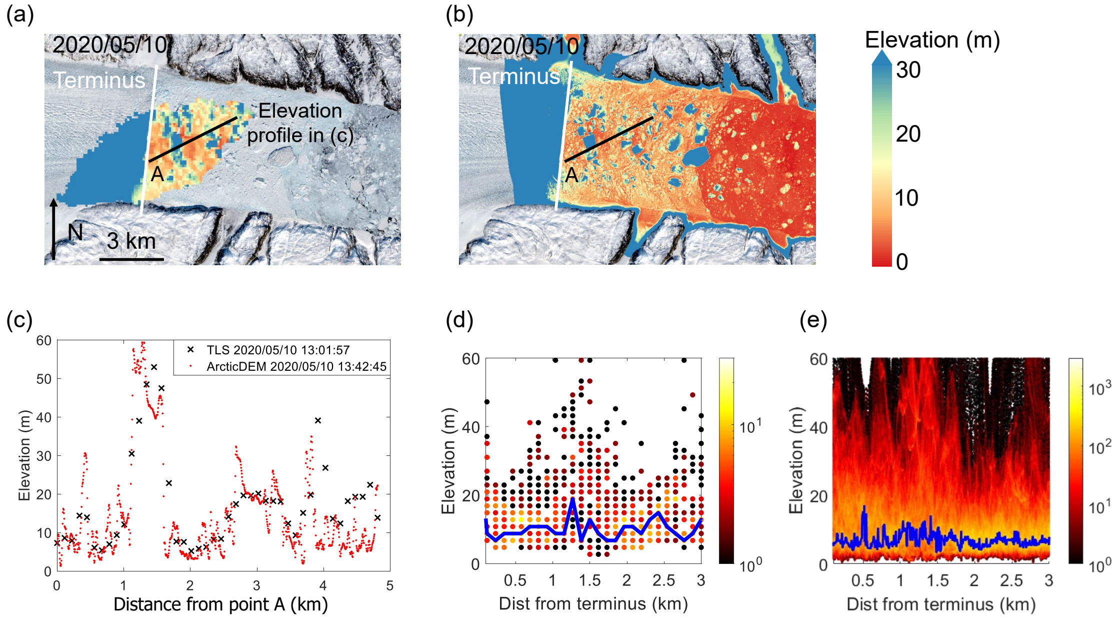

Supplementary Figure 13: A comparison of mélange surface elevation from TLS and ArcticDEM at Helheim Glacier. (a), (b) are TLS, ArcticDEM measured elevation map after accounting for local differences between the ellipsoid and geoid [12] with tidal corrections [9], overlain on a Sentinel-2 image (acquired on 10 May 2020). Images are in polar stereographic projection (EPSG: 3413). (c) Surface elevation profiles extracted along the black line in (a)-(b). Black cross markers indicate data acquired from TLS on 10 May 2020, 13:01:57 UTC, and red dot markers indicate data acquired from ArcticDEM on 10 May 2020, 13:42:45 UTC. The horizontal axis shows the distance from point A in (a). (d), (e) are TLS and ArcticDEM measured surface elevation profiles for the mélange displayed as density plots; the color bar denotes the number of data points that have the same elevation and distance from terminus values. For any specific distance from terminus, we find the elevation value that has the maximum number of data points. Solid blue lines connect these elevation values along the distance from terminus as the representative mélange elevation profiles.

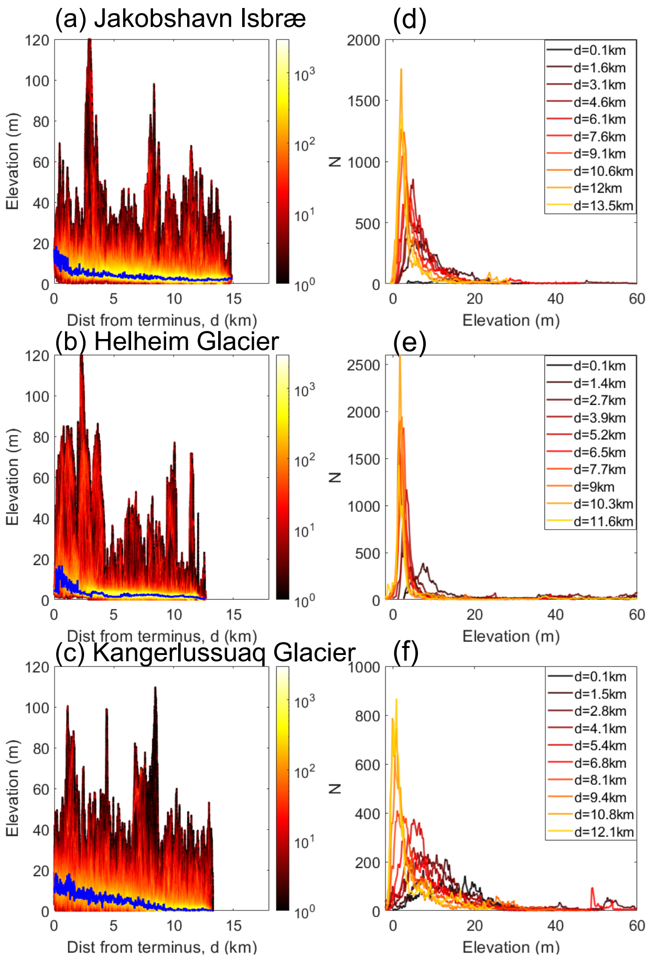

Supplementary Figure 14: Digital elevation models of (a) Jakobshavn Isbræ (19 Apr 2014), (b) Helheim Glacier (30 Mar 2014), and (c) Kangerlussuaq Glacier (5 May 2020) from the ArcticDEM, after accounting for the local difference between the ellipsoid and the geoid. Surface elevation profiles for the mélange displayed as density plots (14,512,132 $\sim$ 17,108,477 data points in total); the colour bar denotes the number of data points that have the same elevation and distance from terminus values. In (d)-(f), we plot the number of data points against the surface elevation value at 10 distances from terminus. For any specific distance from terminus, we find the elevation value that has the maximum number of data points. Solid blue lines connect these elevations values along the distance from terminus to represent the averaged mélange elevation profiles.

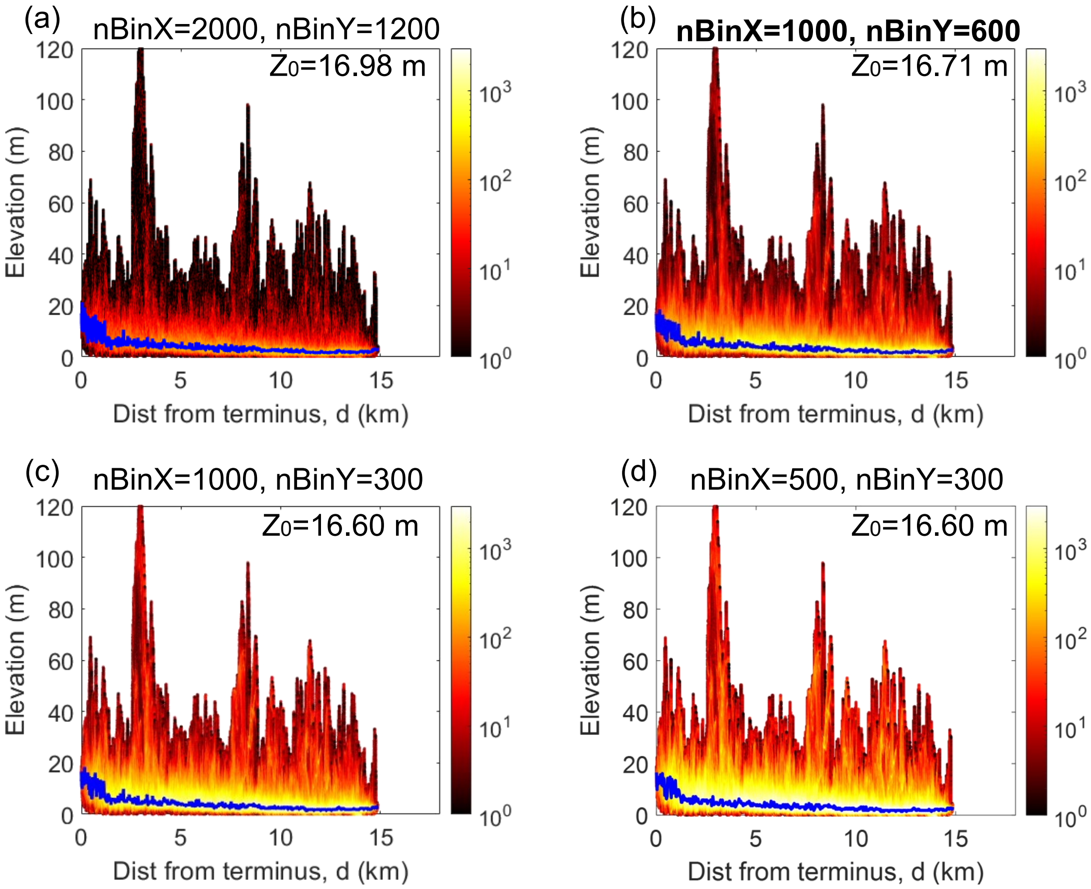

Supplementary Figure 15: Digital elevation models of Jakobshavn Isbræ (19 Apr 2014) from the ArcticDEM, after accounting for the local difference between the ellipsoid and the geoid. Surface elevation profiles for the mélange displayed as density plots (17,108,477 data points); the colour bar denotes the number of data points that have the same elevation and distance from terminus values. We vary the number of horizontal and vertical bins and produce four point density plots (a)-(d). Solid blue lines present the averaged mélange elevation profiles, which turn out to be insensitive to the choice of number of bins. The difference in mélange elevation at the terminus (Z_0) is only 0.3 m. For all ArcticDEM point density plots in the main text, we adopt parameters shown in (b): 1000 horizontal bins and 600 vertical bins.

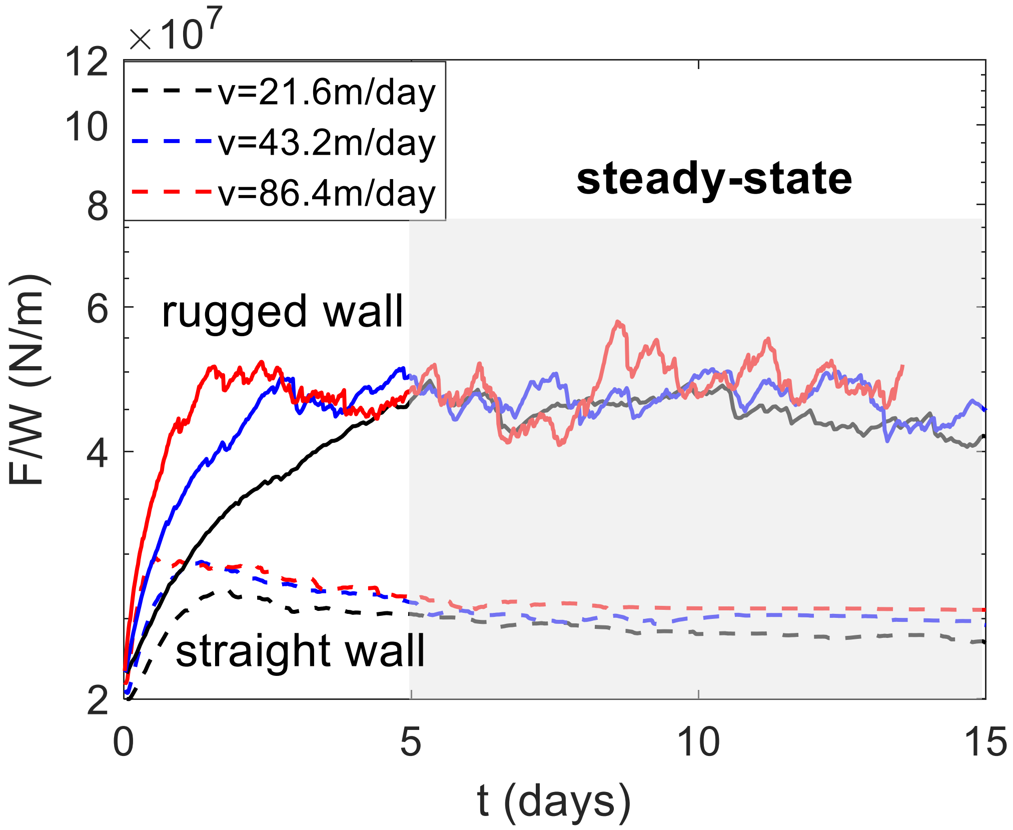

Supplementary Figure 16: The temporal evolution of F/W during the terminus motion for straight (dashed lines) and rugged (solid lines) fjord walls. The initial thickness of the mélange is 280 m. The red, blue, and black colours correspond to a terminus velocity of 86.4 m/day, 43.2 m/day (adopted for all simulations in the main text), 21.6 m/day, respectively. Simulations reach the steady state after 5 days. The results show that the averaged steady-state buttressing force is invariant to the terminus velocity.

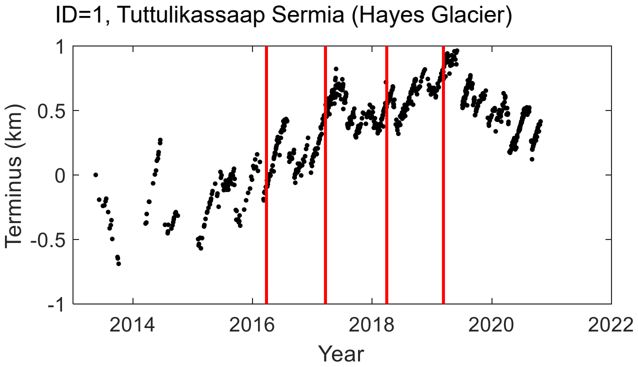

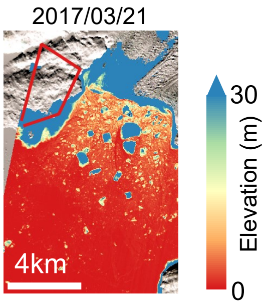

Terminus advance

Date	Z0 (m)
2016/03/26	10.0
**2017/03/21**	**16.7**
2018/03/30	5.8
2019/03/11	6.7

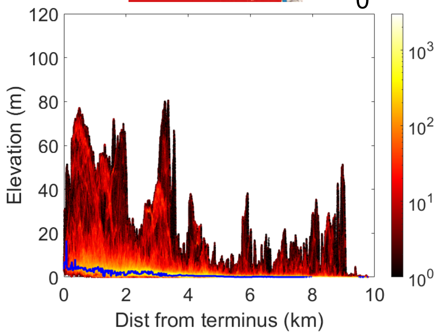

Supplementary Figure 17: A time series of terminus position [11], ArcticDEM acquisition dates and observed mélange freeboard heights (Z_0) at the terminus of Tuttulikassaap Sermia (Hayes Glacier). Solid red lines mark the dates with terminus advances and dashed black lines mark the dates with terminus retreats. The top-right image shows the mélange elevation above mean sea level from a specific ArcticDEM strip from one of the acquisition dates (highlighted in the bottom-left table), overlain on satellite image acquired around the same date. The image is in polar stereographic projection (EPSG: 3413). For co-registration to improve the vertical accuracy, we calculated averaged elevation offsets between individual DEM strips and the mosaic DEM (registered with ICESat-2) along line segments on the rock marked as red solid lines. The bottom-right image shows the surface elevation profile for the mélange displayed as a density plot. The solid blue line is the representative mélange elevation profile.

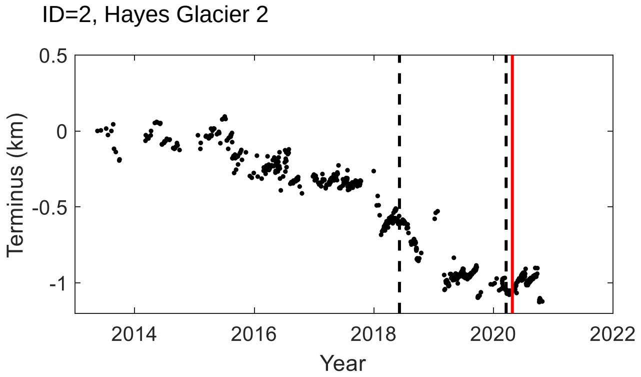

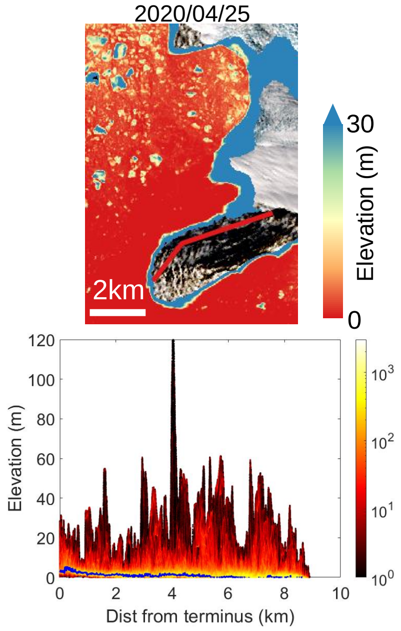

Terminus advance

Date	Z0 (m)
**2020/04/25**	**5.6**

Terminus retreat

Date	Z0 (m)
2018/06/05	3.7
2020/03/18	2.8

Supplementary Figure 18: Same legends as in Supplementary Fig. 17 but at the terminus of Hayes Glacier 2.

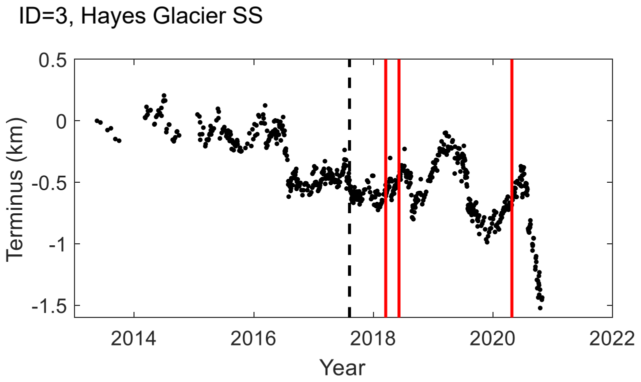

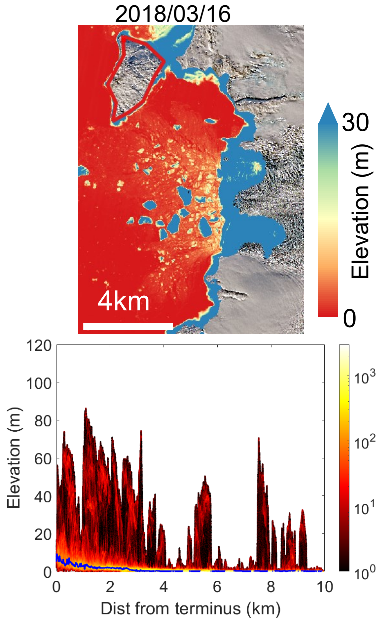

Terminus advance

Date	Z0 (m)
**2018/03/16**	**9.2**
2018/06/05	8.2
2020/04/25	8.3

Terminus retreat

Date	Z0 (m)
2017/08/07	3.4

Supplementary Figure 19: Same legends as in Supplementary Fig. 17 but at the terminus of Hayes Glacier SS.

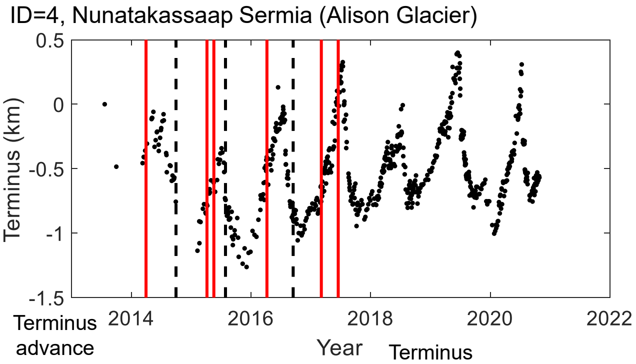

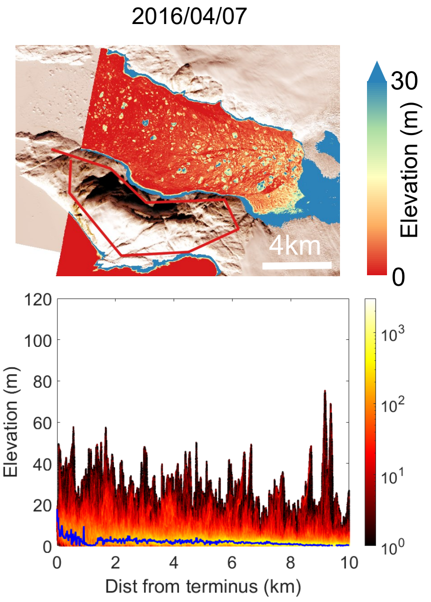

Terminus advance

Date	Z_0 (m)
2014/03/30	17.2
2015/04/06	11.1
2015/05/18	15.2
**2016/04/07**	**17.9**
2017/03/05	15.5
2017/06/16	14.2

Terminus retreat

Date	Z_0 (m)
2014/09/29	1.2
2015/07/28	1.1
2016/09/14	2.2

Supplementary Figure 20: Same legends as in Supplementary Fig. 17 but at the terminus of Nunatakassaap Sermia (Alison Glacier).

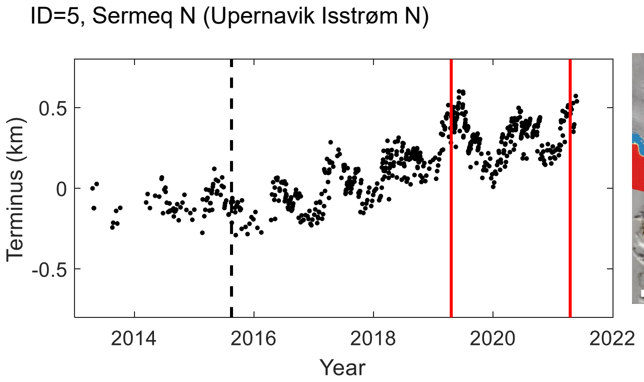

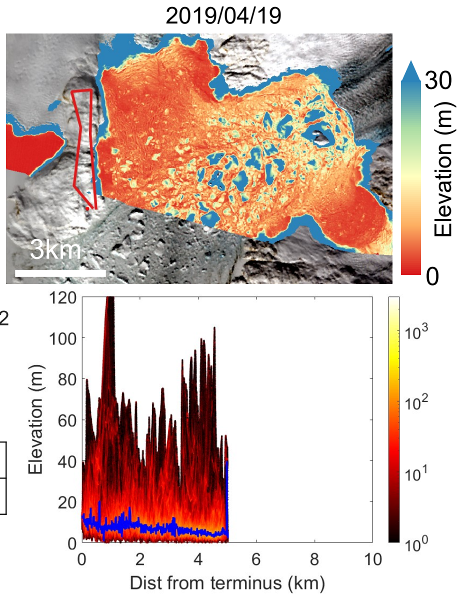

Terminus advance

Date	Z_0 (m)
**2019/04/19**	**17.0**
2021/04/15	10.7

Terminus retreat

Date	Z_0 (m)
2015/08/16	1.4

Supplementary Figure 21: Same legends as in Supplementary Fig. 17 but at the terminus of Sermeq N (Upernavik Isstrøm N).

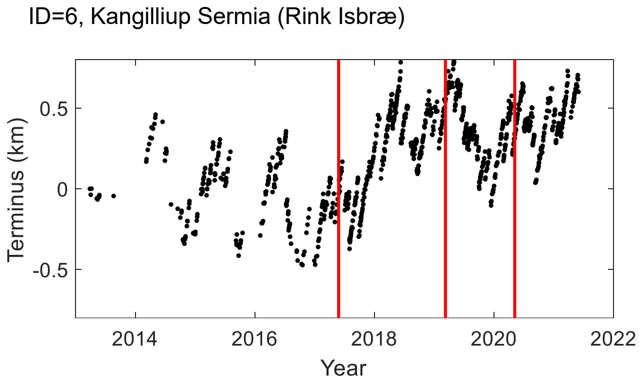

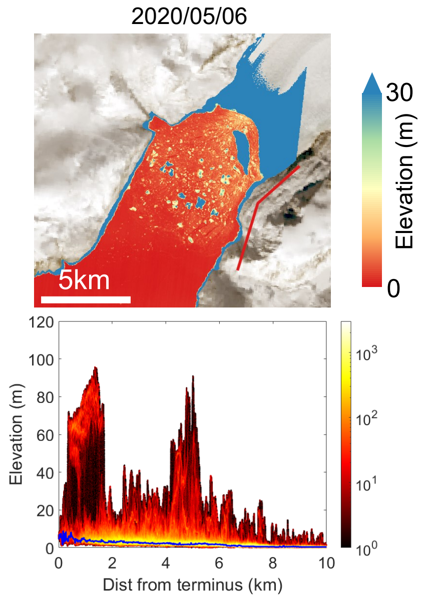

Terminus advance

Date	Z0 (m)
2017/05/26	5.8
2019/03/09	7.6
**2020/05/06**	**9.1**

Supplementary Figure 22: Same legends as in Supplementary Fig. 17 but at the terminus of Kangilliup Sermia (Rink Isbræ).

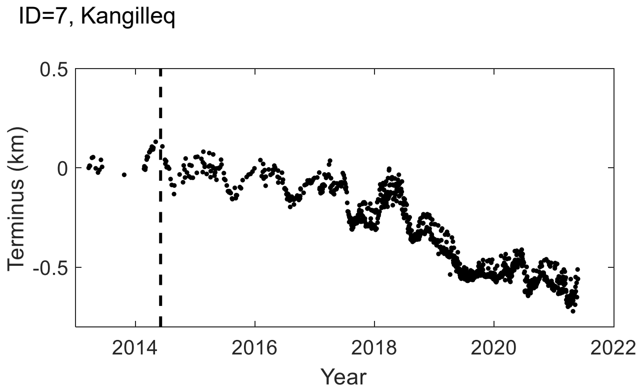

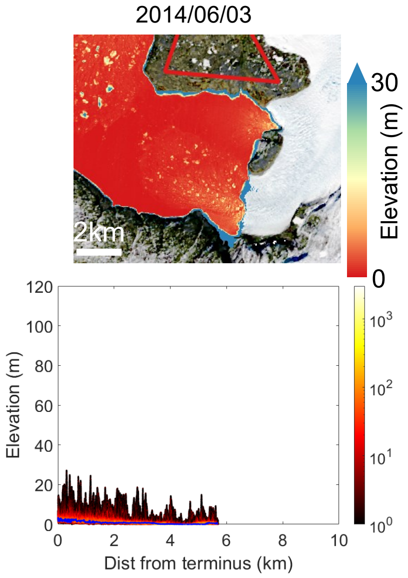

Terminus retreat

Date	Z0 (m)
**2014/06/03**	**2.8**

Supplementary Figure 23: Same legends as in Supplementary Fig. 17 but at the terminus of Kangilleq.

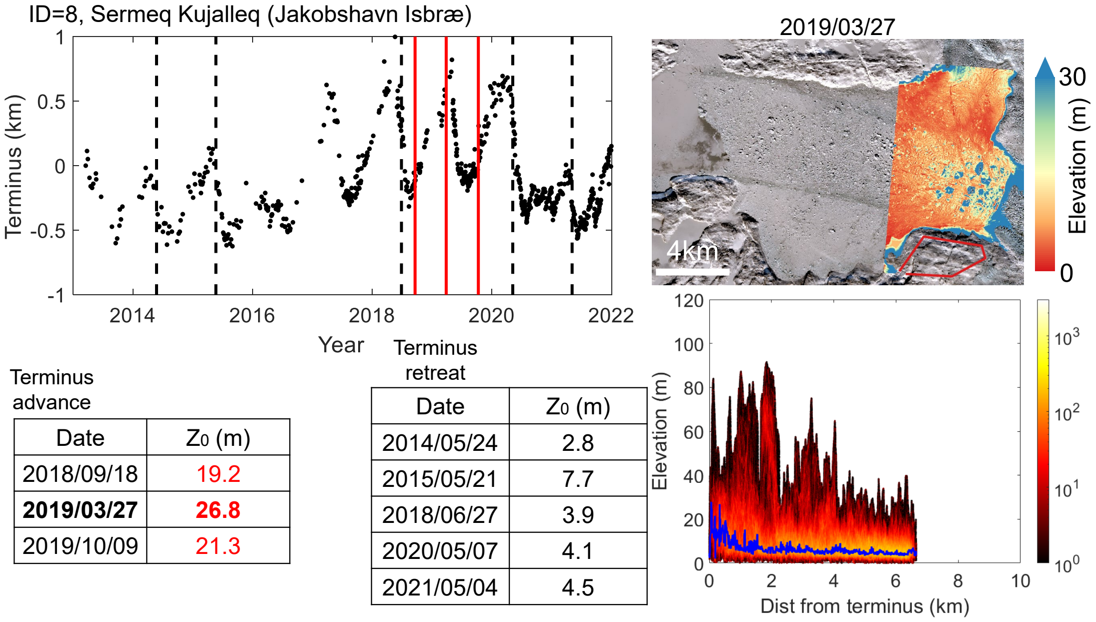

Terminus advance

Date	Z0 (m)
2018/09/18	19.2
**2019/03/27**	**26.8**
2019/10/09	21.3

Terminus retreat

Date	Z0 (m)
2014/05/24	2.8
2015/05/21	7.7
2018/06/27	3.9
2020/05/07	4.1
2021/05/04	4.5

Supplementary Figure 24: Same legends as in Supplementary Fig. 17 but at the terminus of Sermeq Kujalleq (Jakobshavn Isbræ).

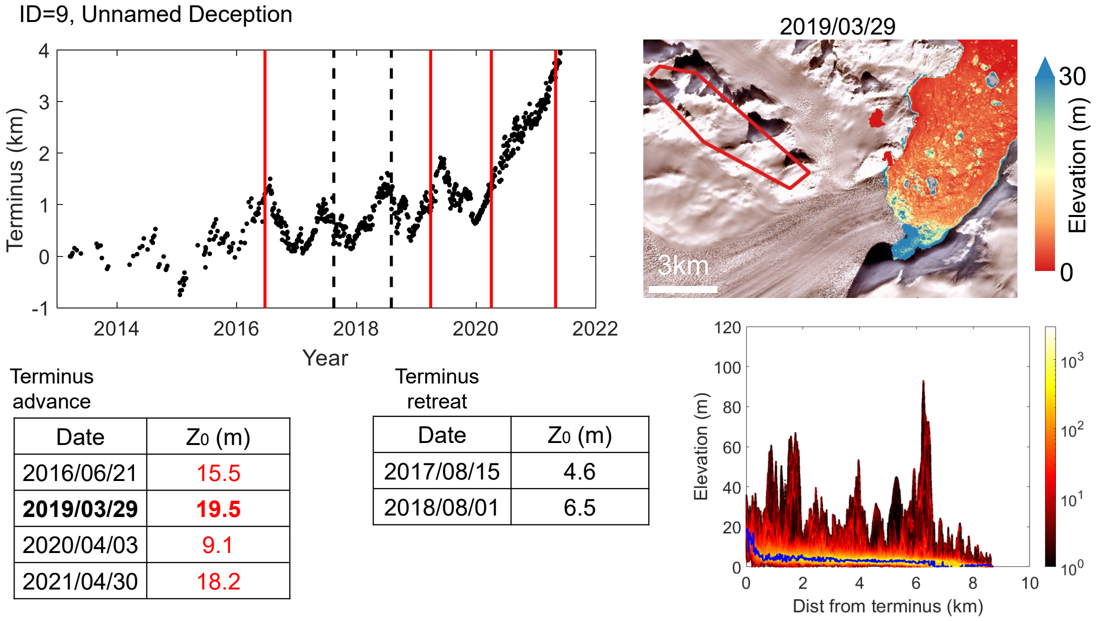

Terminus advance

Date	Z0 (m)
2016/06/21	15.5
**2019/03/29**	**19.5**
2020/04/03	9.1
2021/04/30	18.2

Terminus retreat

Date	Z0 (m)
2017/08/15	4.6
2018/08/01	6.5

Supplementary Figure 25: Same legends as in Supplementary Fig. 17 but at the terminus of Unnamed Deception.

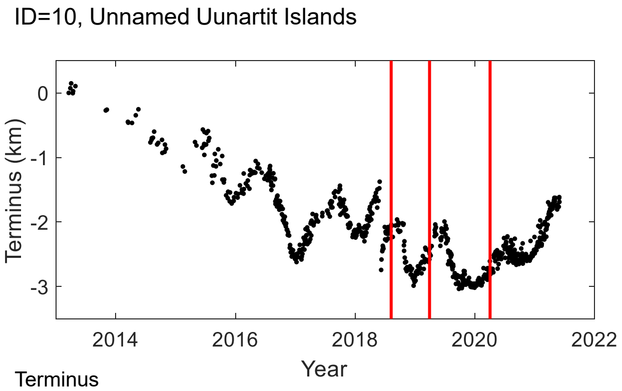

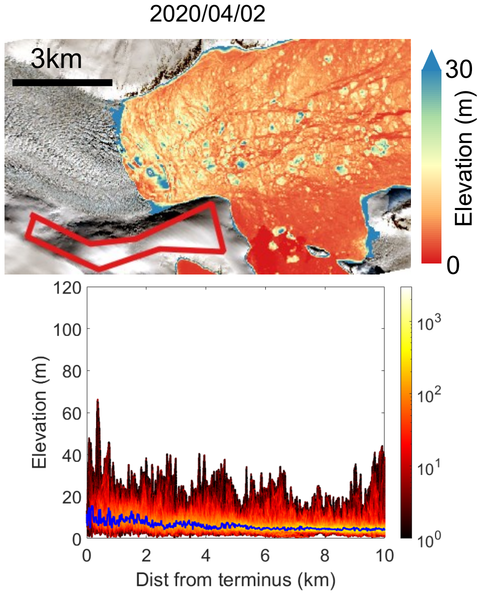

Terminus advance

Date	Z_0 (m)
2018/08/07	10.3
2019/03/29	8.8
**2020/04/02**	**15.7**

Supplementary Figure 26: Same legends as in Supplementary Fig. 17 but at the terminus of Unnamed Uunartit Islands.

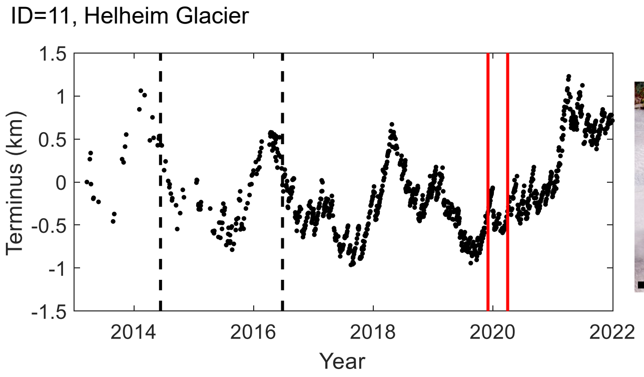

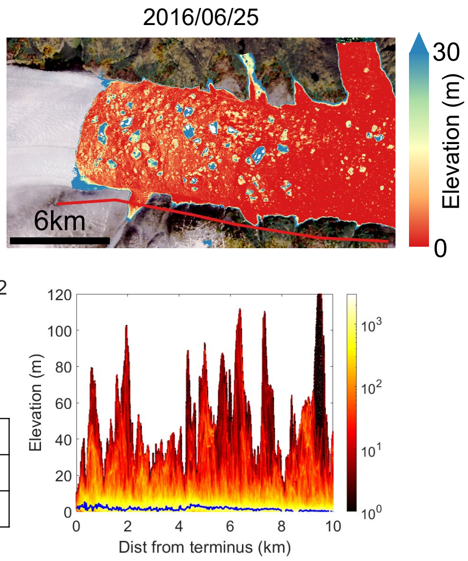

Terminus advance

Date	Z_0 (m)
2019/11/30	16.2
2020/03/30	15.0

Terminus retreat

Date	Z_0 (m)
2014/06/11	5.2
**2016/06/25**	**5.6**

Supplementary Figure 27: Same legends as in Supplementary Fig. 17 but at the terminus of Helheim Glacier. The two mélange freeboard heights (Z_0) acquired on 30 Nov 2019 and 30 Mar 2020 come from LiDAR data presented in section 1.1 in the main text.

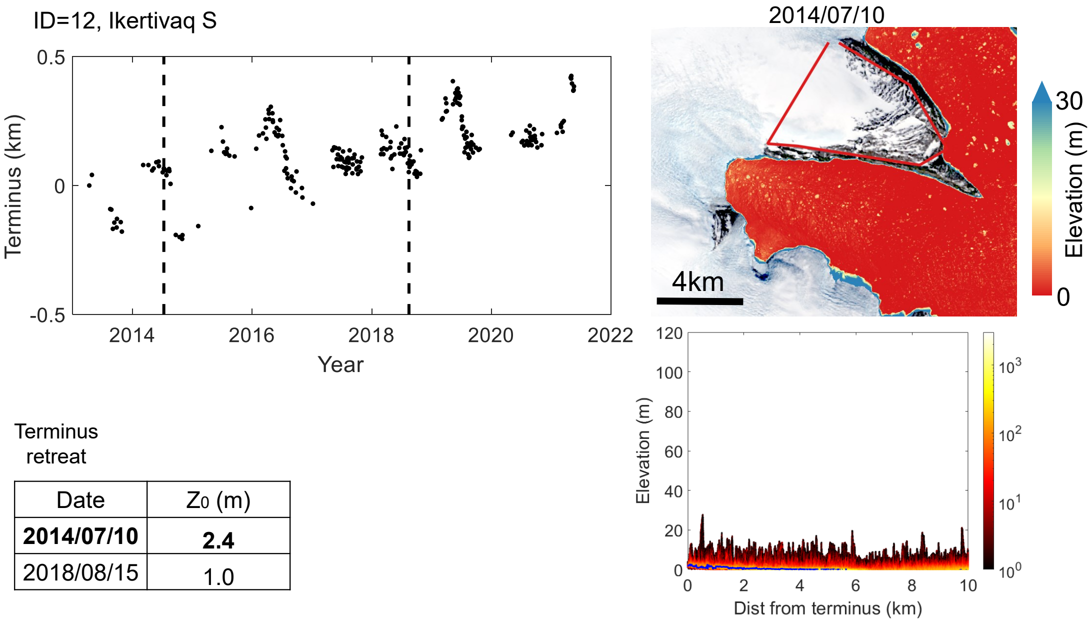

Date	Z0 (m)
**2014/07/10**	**2.4**
2018/08/15	1.0

Supplementary Figure 28: Same legends as in Supplementary Fig. 17 but at the terminus of Ikertivaq S.

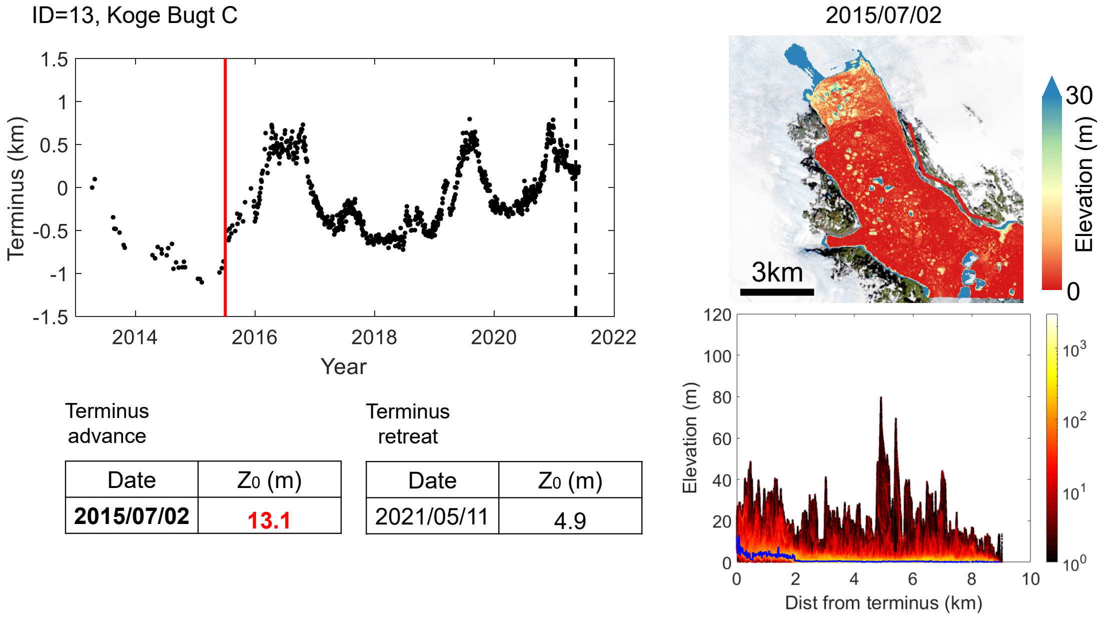

Date	Z0 (m)
**2015/07/02**	**13.1**

Date	Z0 (m)
2021/05/11	4.9

Supplementary Figure 29: Same legends as in Supplementary Fig. 17 but at the terminus of Koge Bugt C.

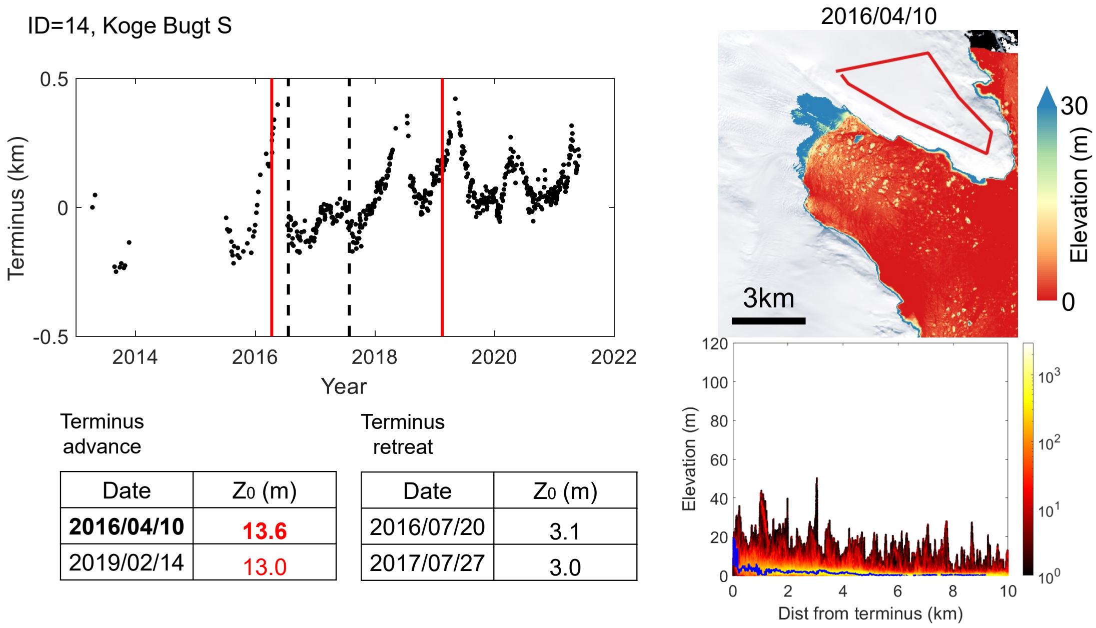

Date	Z0 (m)
**2016/04/10**	**13.6**
2019/02/14	13.0

Date	Z0 (m)
2016/07/20	3.1
2017/07/27	3.0

Supplementary Figure 30: Same legends as in Supplementary Fig. 17 but at the terminus of Koge Bugt S.

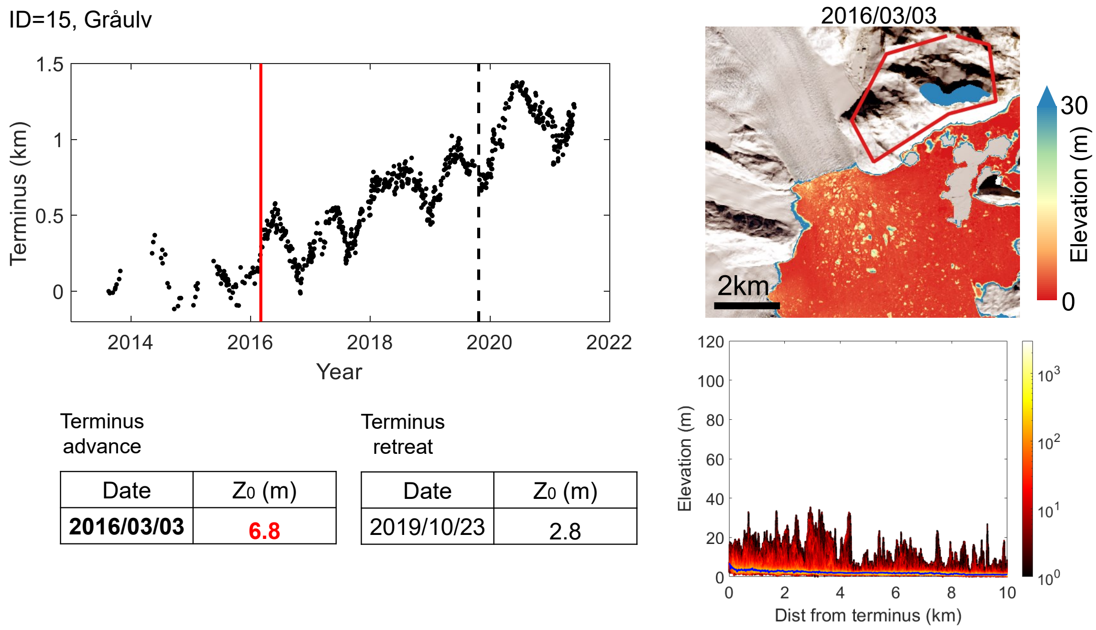

Date	Z0 (m)
**2016/03/03**	**6.8**

Date	Z0 (m)
2019/10/23	2.8

Supplementary Figure 31: Same legends as in Supplementary Fig. 17 but at the terminus of Gråulv.

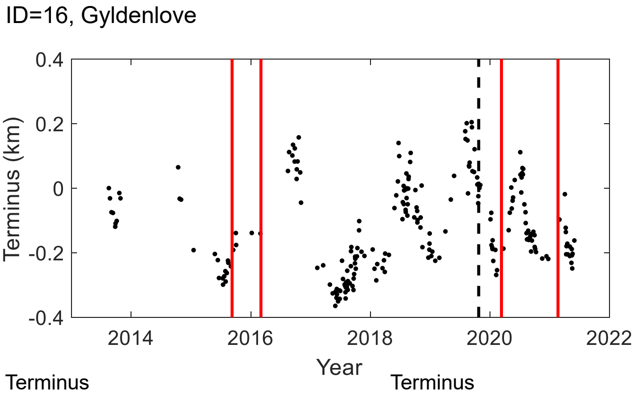

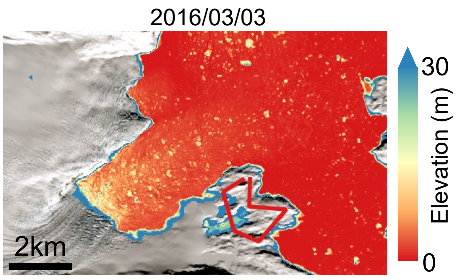

Terminus advance

Date	Z₀ (m)
2015/09/08	8.0
**2016/03/03**	**12.9**
2020/03/11	7.9
2021/02/19	10.5

Terminus retreat

Date	Z₀ (m)
2019/10/23	4.0

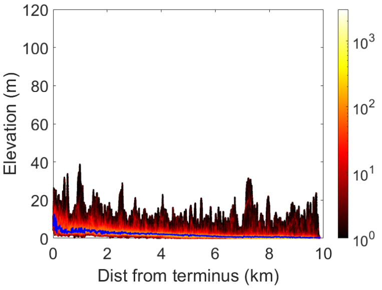

Supplementary Figure 32: Same legends as in Supplementary Fig. 17 but at the terminus of Gyldenlove.

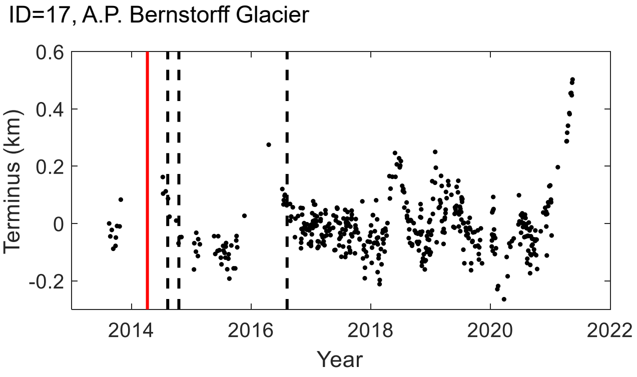

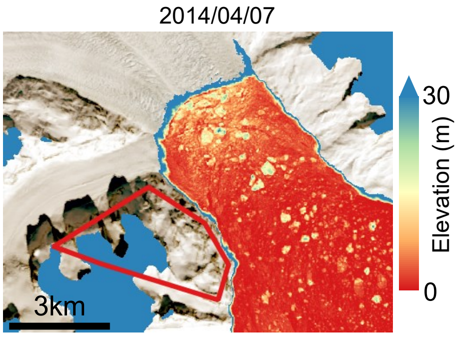

Terminus advance

Date	Z₀ (m)
**2014/04/07**	**15.1**

Terminus retreat

Date	Z₀ (m)
2014/08/09	4.6
2014/10/16	4.4
2016/08/07	7.0

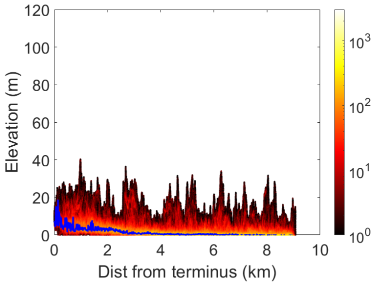

Supplementary Figure 33: Same legends as in Supplementary Fig. 17 but at the terminus of A.P. Bernstorff Glacier.

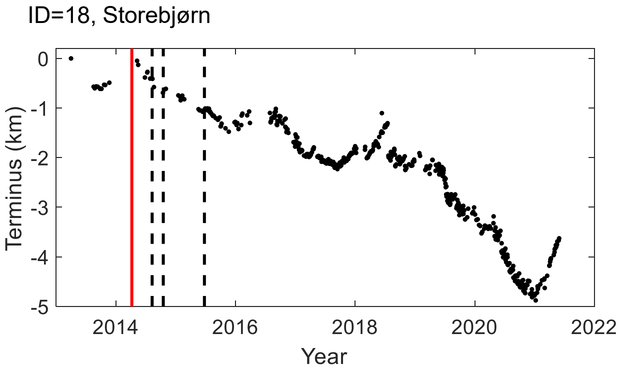

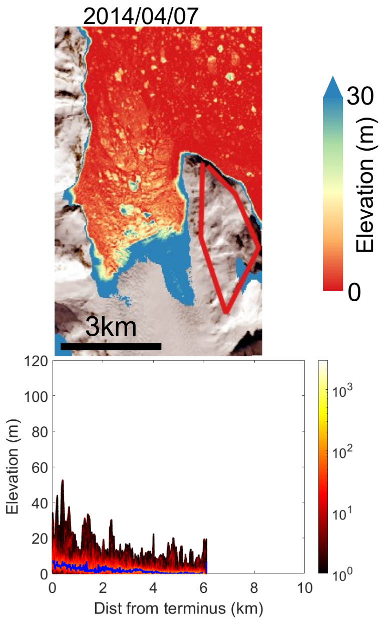

Terminus advance

Date	Z0 (m)
**2014/04/07**	**7.2**

Terminus retreat

Date	Z0 (m)
2014/08/09	5.7
2014/10/16	6.2
2015/06/24	0.2

Supplementary Figure 34: Same legends as in Supplementary Fig. 17 but at the terminus of Storebjørn.

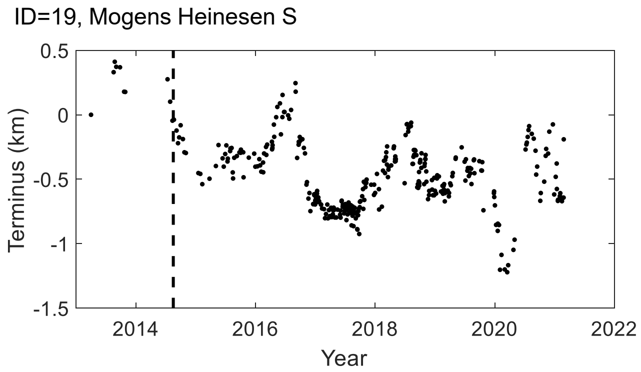

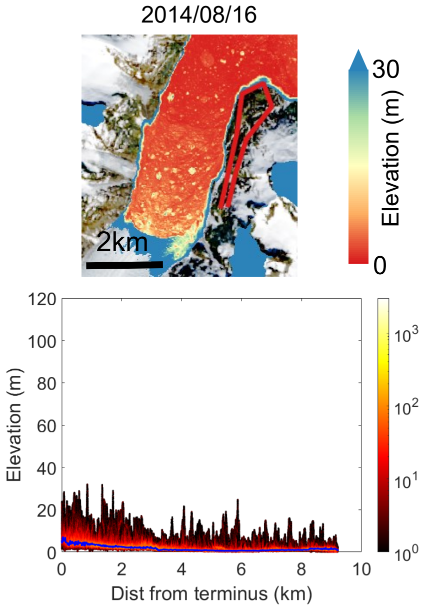

Terminus retreat

Date	Z0 (m)
**2014/08/16**	**7.1**

Supplementary Figure 35: Same legends as in Supplementary Fig. 17 but at the terminus of Mogens Heinesen S.

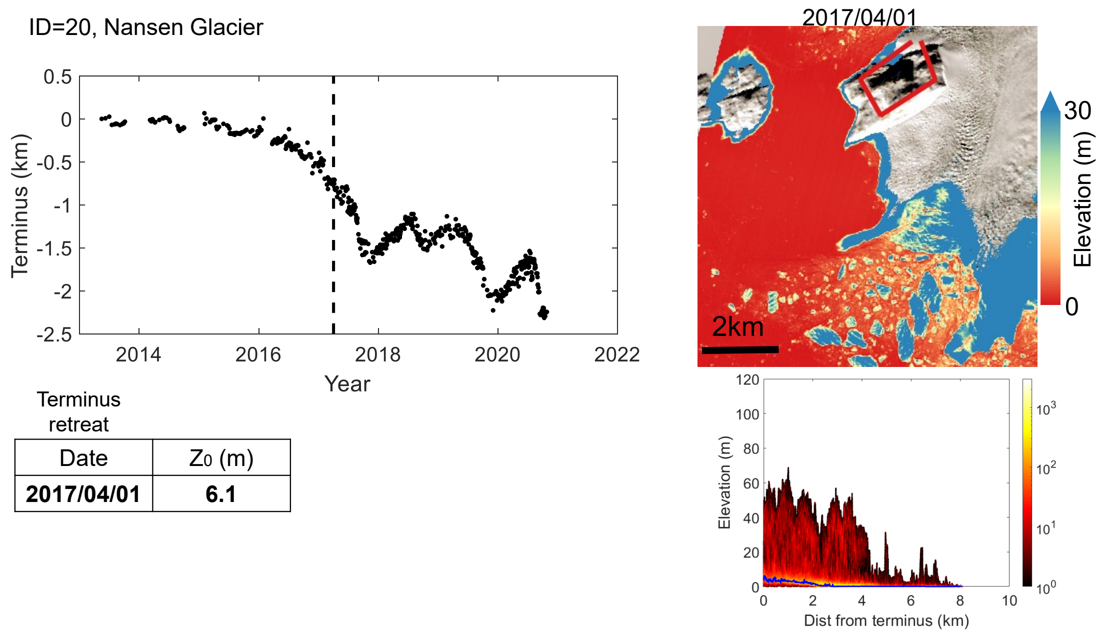

Date	Z0 (m)
**2017/04/01**	**6.1**

Supplementary Figure 36: Same legends as in Supplementary Fig. 17 but at the terminus of Nansen Glacier.

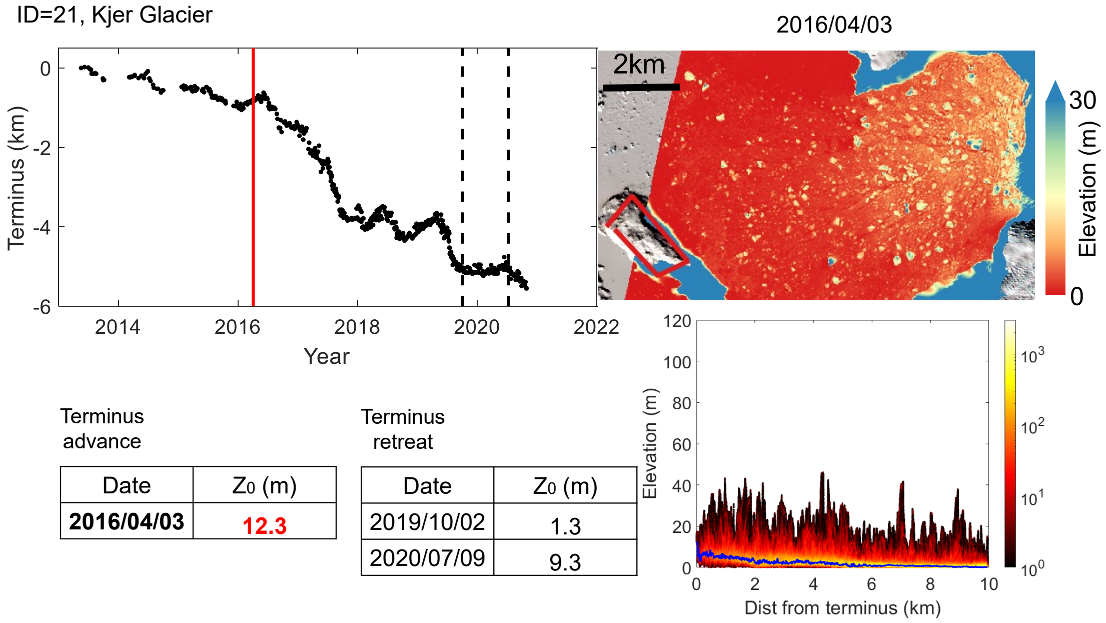

Date	Z0 (m)
**2016/04/03**	**12.3**

Date	Z0 (m)
2019/10/02	1.3
2020/07/09	9.3

Supplementary Figure 37: Same legends as in Supplementary Fig. 17 but at the terminus of Kjer Glacier.

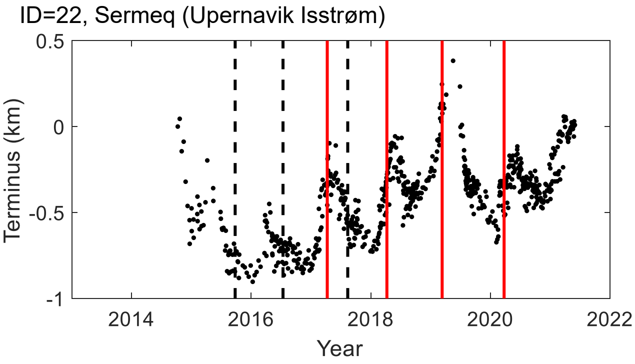

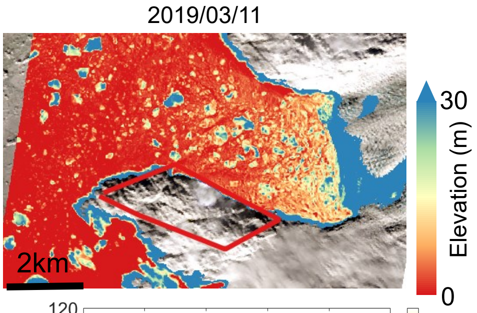

Terminus advance

Date	Z0 (m)
2017/04/09	23.6
2018/04/08	11.4
**2019/03/11**	**26.5**
2020/03/24	14.0

Terminus retreat

Date	Z0 (m)
2015/09/24	2.2
2016/07/13	2.8
2017/08/12	2.1

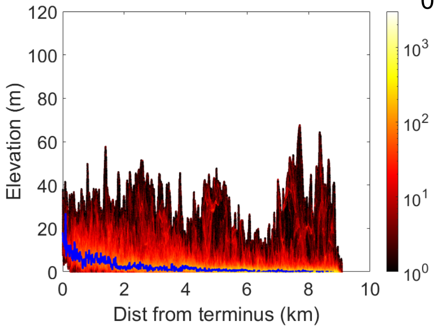

Supplementary Figure 38: Same legends as in Supplementary Fig. 17 but at the terminus of Sermeq (Upernavik Isstrøm).

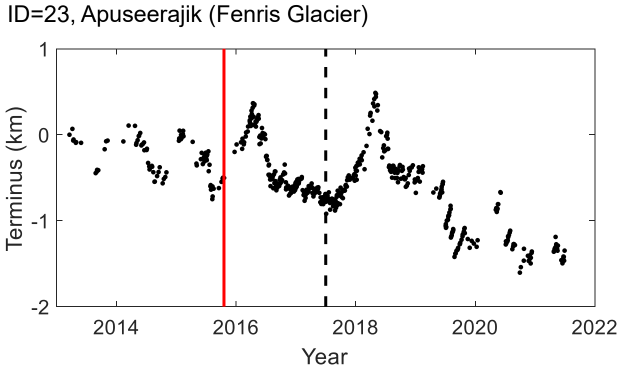

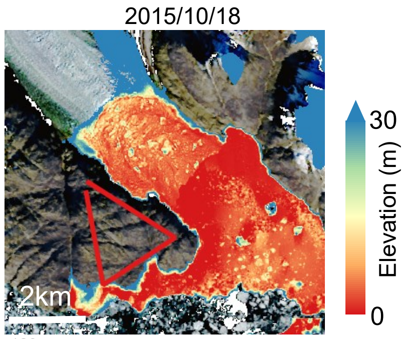

Terminus advance

Date	Z0 (m)
**2015/10/18**	**11.7**

Terminus retreat

Date	Z0 (m)
2017/07/01	2.4

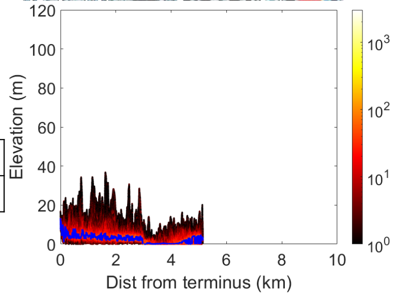

Supplementary Figure 39: Same legends as in Supplementary Fig. 17 but at the terminus of Apuseerajik (Fenris Glacier).

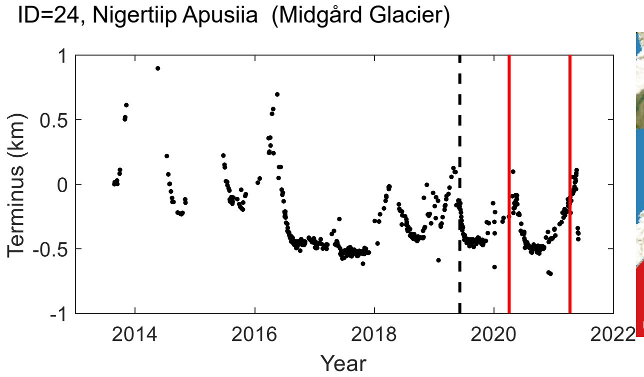

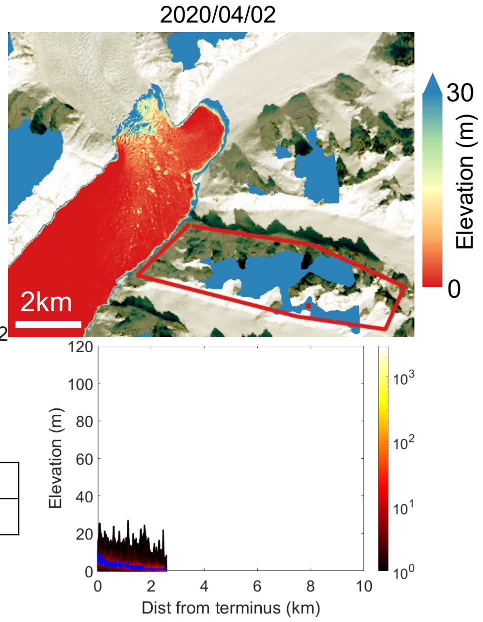

Terminus advance

Date	Z0 (m)
**2020/04/02**	**9.9**
2021/04/08	7.6

Terminus retreat

Date	Z0 (m)
2019/06/05	3.0

Supplementary Figure 40: Same legends as in Supplementary Fig. 17 but at the terminus of Nigertiip Apusiia (Midgård Glacier).

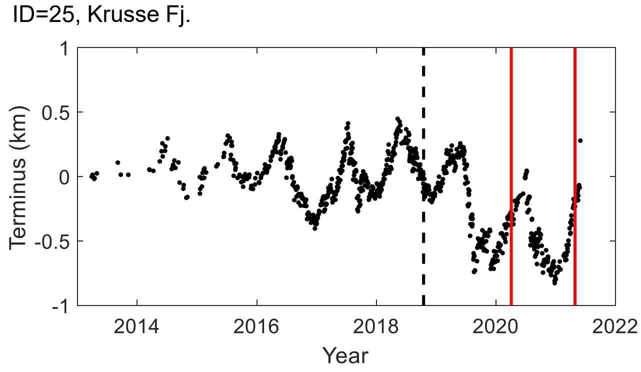

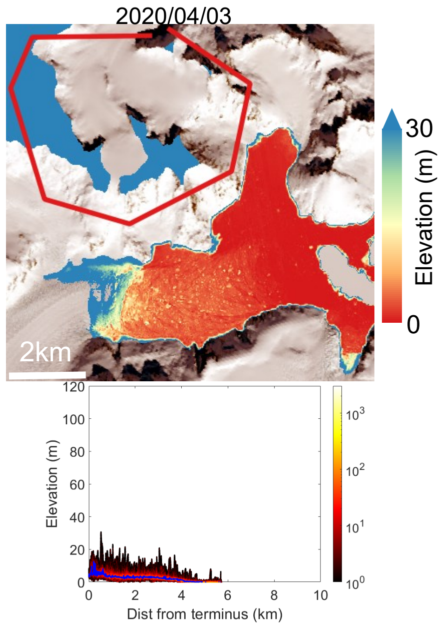

Terminus advance

Date	Z0 (m)
**2020/04/03**	**11.5**
2021/04/27	8.5

Terminus retreat

Date	Z0 (m)
2018/10/15	0.1

Supplementary Figure 41: Same legends as in Supplementary Fig. 17 but at the terminus of Krusse Fj.

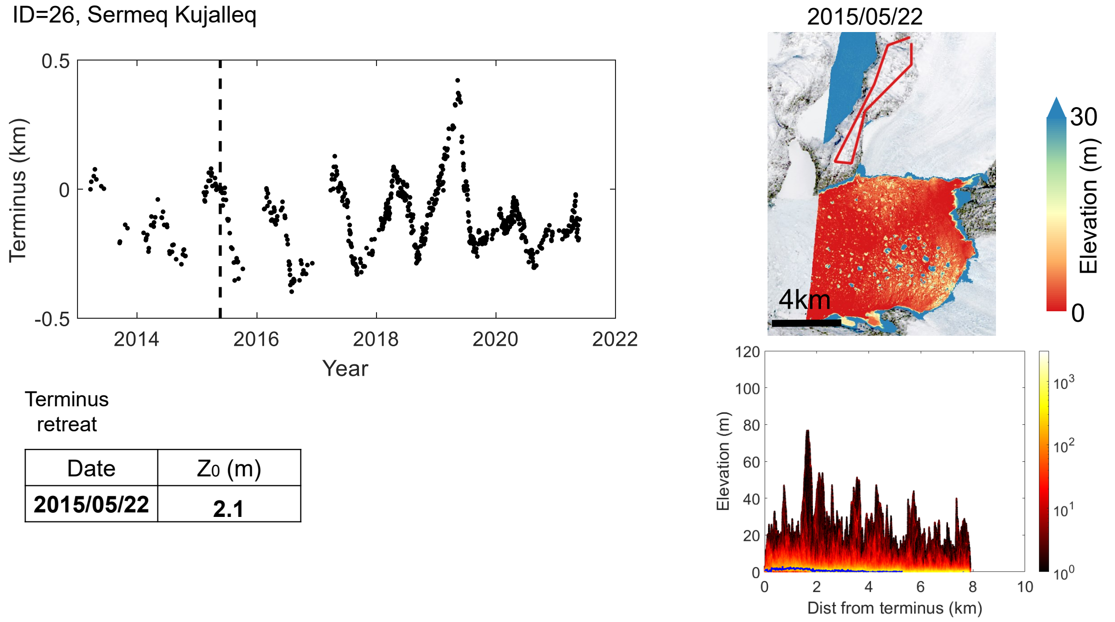

Date	Z_0 (m)
**2015/05/22**	**2.1**

Supplementary Figure 42: Same legends as in Supplementary Fig. 17 but at the terminus of Sermeq Kujalleq.

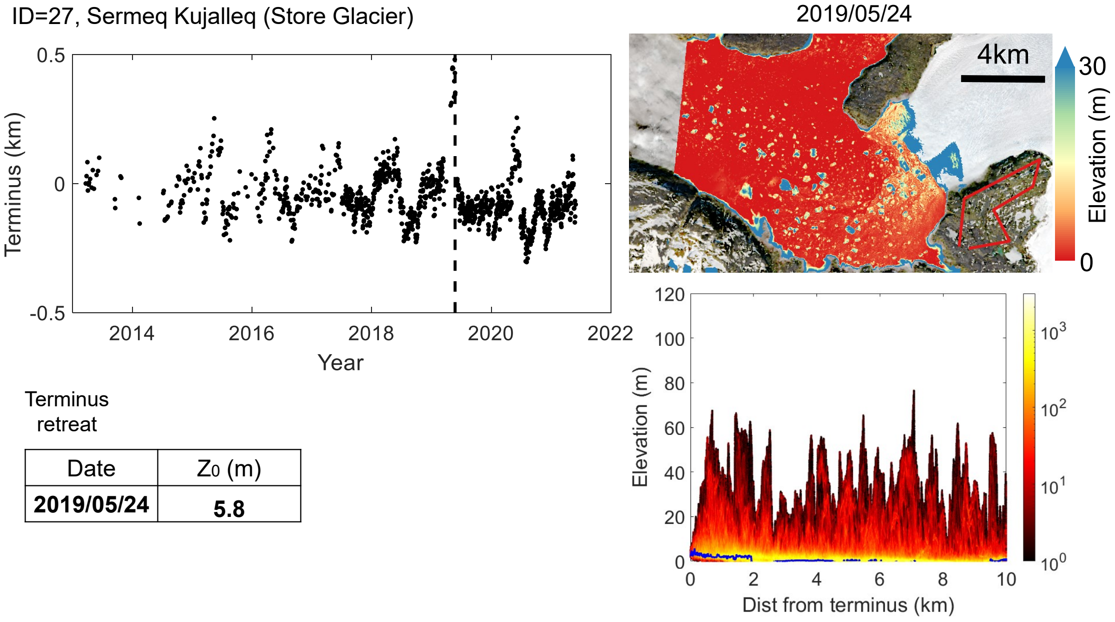

Date	Z_0 (m)
**2019/05/24**	**5.8**

Supplementary Figure 43: Same legends as in Supplementary Fig. 17 but at the terminus of Sermeq Kujalleq (Store Glacier).

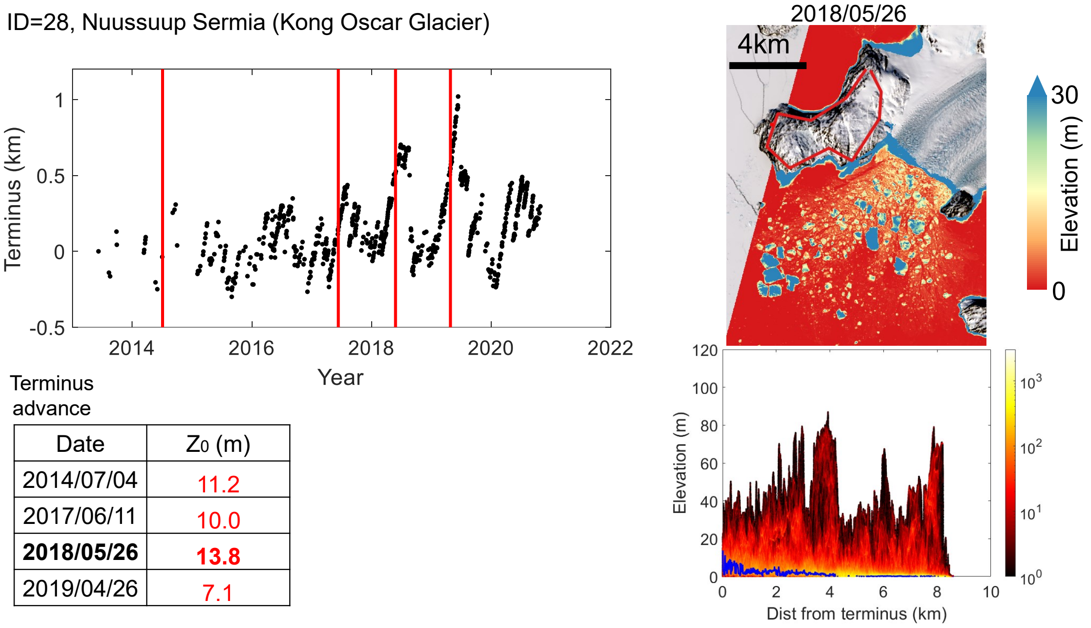

Supplementary Figure 44: Same legends as in Supplementary Fig. 17 but at the terminus of Nuussuup Sermia (Kong Oscar Glacier).

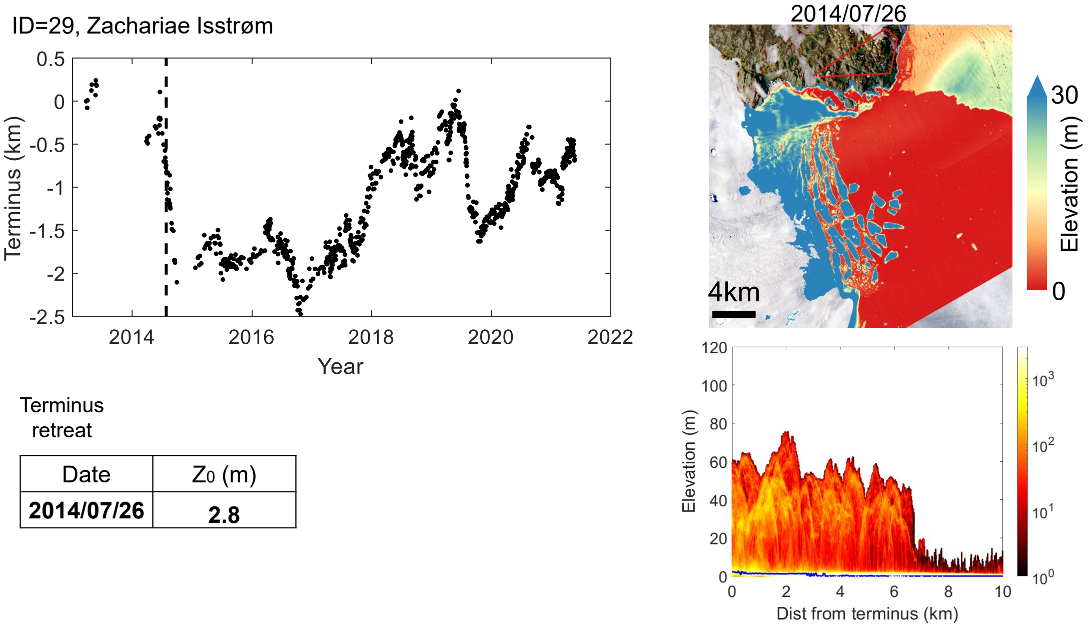

Supplementary Figure 45: Same legends as in Supplementary Fig. 17 but at the terminus of Zachariae Isstrøm.

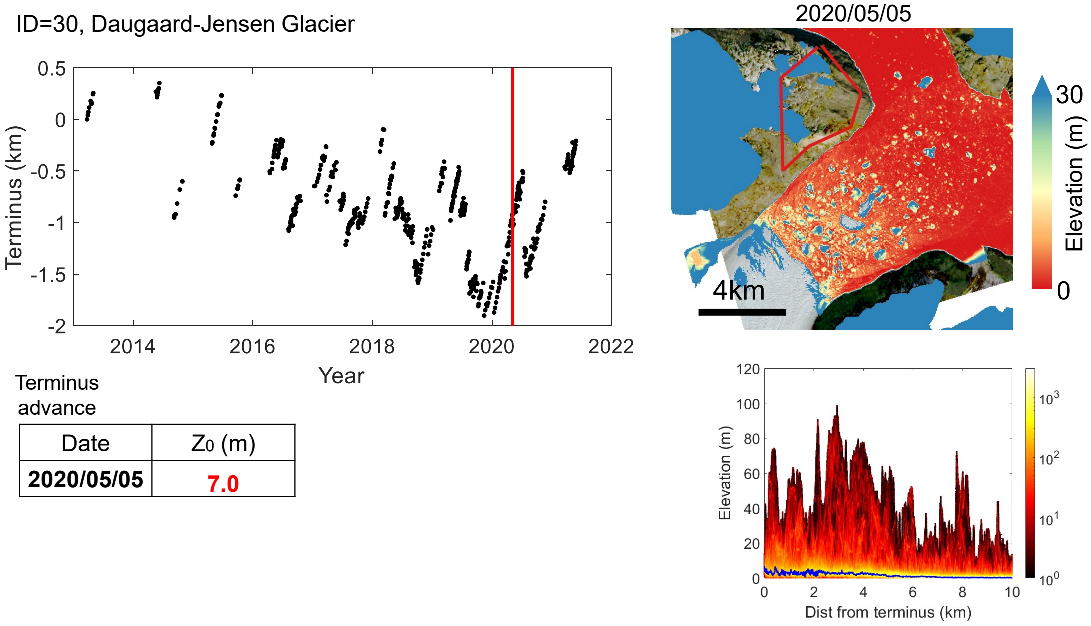

Date	Z0 (m)
**2020/05/05**	**7.0**

Supplementary Figure 46: Same legends as in Supplementary Fig. 17 but at the terminus of Daugaard-Jensen Glacier.

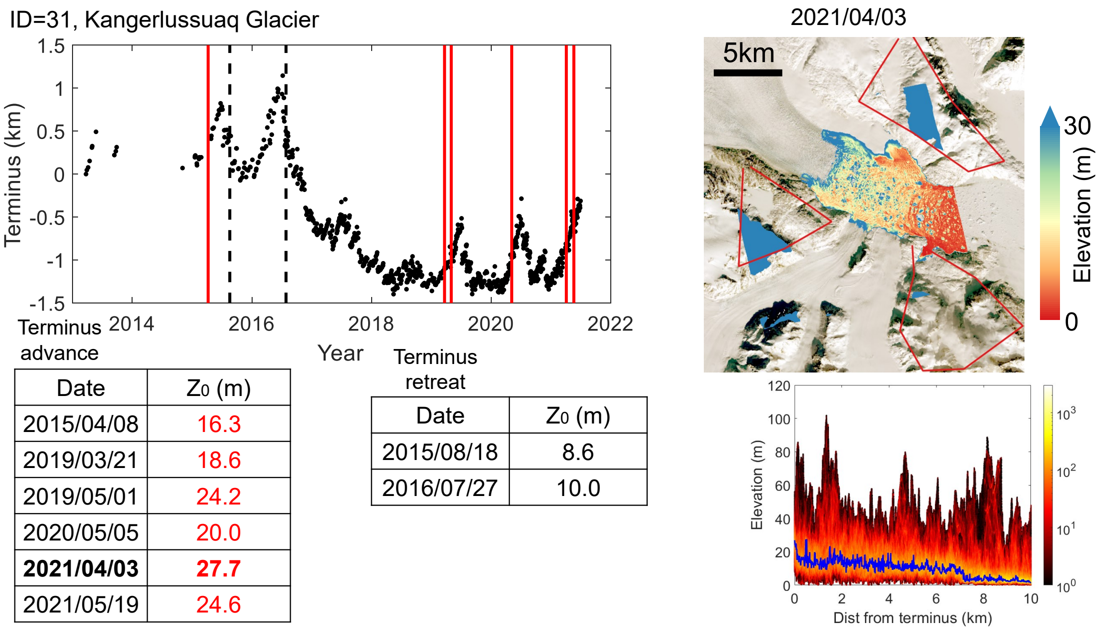

Terminus advance

Date	Z0 (m)
2015/04/08	16.3
2019/03/21	18.6
2019/05/01	24.2
2020/05/05	20.0
**2021/04/03**	**27.7**
2021/05/19	24.6

Terminus retreat

Date	Z0 (m)
2015/08/18	8.6
2016/07/27	10.0

Supplementary Figure 47: Same legends as in Supplementary Fig. 17 but at the terminus of Kangerlussuaq Glacier.

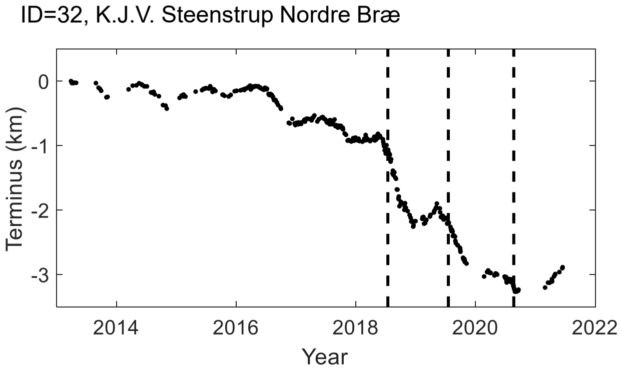

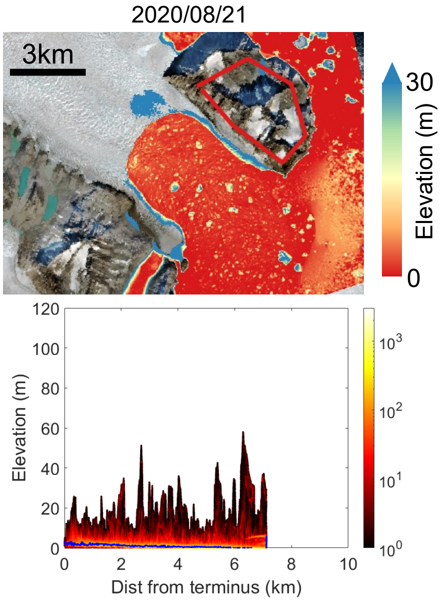

Terminus retreat

Date	Z_0 (m)
2018/07/14	0.8
2019/07/18	2.1
**2020/08/21**	**3.2**

Supplementary Figure 48: Same legends as in Supplementary Fig. 17 but at the terminus of K.J.V. Steenstrup Nordre Bræ.

Supplementary References

[1] MacMinn, C. W., Dufresne, E. R. & Wettlaufer, J. S. Fluid-driven deformation of a soft granular material. *Physical Review X* **5**, 011020 (2015).

[2] Auton, L. C. & MacMinn, C. W. Large poroelasto-plastic deformations due to radially outward fluid injection. *Journal of the Mechanics and Physics of Solids* **132**, 103690 (2019).

[3] Meng, Y., Li, W. & Juanes, R. Crossover from viscous fingering to fracturing in cohesive wet granular media: a photoporomechanics study. *Soft Matter* **19**, 7136–7148 (2023).

[4] MacAyeal, D. R. Large-scale ice flow over a viscous basal sediment: Theory and application to ice stream B, Antarctica. *Journal of Geophysical Research: Solid Earth* **94**, 4071–4087 (1989).

[5] Burton, J. C., Amundson, J. M., Cassotto, R., Kuo, C.-C. & Dennin, M. Quantifying flow and stress in ice mélange, the world's largest granular material. *Proceedings of the National Academy of Sciences* **115**, 5105–5110 (2018).

[6] Favier de Coulomb, A., Bouzid, M., Claudin, P., Clément, E. & Andreotti, B. Rheology of granular flows across the transition from soft to rigid particles. *Physical Review Fluids* **2**, 102301 (2017).

[7] Karim, M. Y. & Corwin, E. I. Eliminating friction with friction: 2D Janssen effect in a friction-driven system. *Physical Review Letters* **112**, 188001 (2014).

[8] Morlighem, M. *et al.* IceBridge BedMachine Greenland, Version 5. Accessed on 11-20-2024 (2022). URL `https://doi.org/10.5067/GMEVBWFLWA7X`.

[9] Howard, S. L. & Padman, L. Gr1kmtm: Greenland 1 kilometer Tide Model (2021).

[10] Amundson, J. M. & Burton, J. Quasi-static granular flow of ice mélange. *Journal of Geophysical Research: Earth Surface* **123**, 2243–2257 (2018).

[11] Zhang, E., Catania, G. & Trugman, D. T. Autoterm: an automated pipeline for glacier terminus extraction using machine learning and a "big data" repository of Greenland glacier termini. *The Cryosphere* **17**, 3485–3503 (2023).

[12] Ch, F. *et al.* EIGEN-6C4 The latest combined global gravity field model including GOCE data up to degree and order 2190 of GFZ Potsdam and GRGS Toulouse. *GFZ Data Services* **10** (2014).
