# Supplementary material for: Seasonal changes of mélange thickness coincide with Greenland calving dynamics
Source: Nat Commun. 2025 Jan 10;16:573. doi: 10.1038/s41467-024-55241-7 (PMC11724111; doi:10.1038/s41467-024-55241-7)
Supplement: Supplementary file 2 — Description of Additional Supplementary Files [file 41467_2024_55241_MOESM2_ESM.pdf]

Description of Additional Supplementary Files

File Name: Supplementary Movie 1
Description: Animation of mélange simulation with an advancing terminus and in Helheim and Kangerlussuaq fjord configurations. Fjord sidewalls and terminus are shaded brown. Cubic icebergs are shaded dark gray in the side view (the top row), and colored by instantaneous particle velocity in the top view (the second row). The third and fourth row shows the particle velocity field averaged over a 1 hr, and 1 day time window, respectively

File Name: Supplementary Movie 2
Description: Animation of mélange simulation with advancing terminus and 60-meter initial mélange thickness in straight and rugged fjord configurations. Fjord sidewall elements are shaded brown, terminus is shaded light gray, open water is colored in dark blue. Cubic icebergs are shaded dark gray in the side view (the top row), and colored by instantaneous particle velocity in the top view (the second row). The third and fourth row shows the particle velocity field averaged over a 1 hr, and 1 day time window, respectively. The bottom row shows the temporal evolution of the buttressing force per unit width.

File Name: Supplementary Movie 3
Description: Animation of mélange simulation with advancing terminus and 378-meter initial mélange thickness in straight and rugged fjord configurations. Fjord sidewall elements are shaded brown, terminus is shaded light gray, open water is colored in dark blue. Cubic icebergs are shaded dark gray in the side view (the top row), and colored by instantaneous particle velocity in the top view (the second row). The third and fourth row shows the particle velocity field averaged over a 1 hr, and 1 day time window, respectively. The bottom row shows the temporal evolution of the buttressing force per unit width.
